# Supplementary material for: Two Functional Axes of Feedback-Enforced PRC2 Recruitment in Mouse Embryonic Stem Cells
Source: Stem Cell Reports. 2020 Aug 6;15(6):1287–300. doi: 10.1016/j.stemcr.2020.07.007 (PMC7724473; doi:10.1016/j.stemcr.2020.07.007)
Supplement: Document S1. Supplemental Experimental Procedures and Figures S1–S5 [file mmc1.pdf]

Stem Cell Reports, Volume *15*

Supplemental Information

Two Functional Axes of Feedback-Enforced PRC2 Recruitment in Mouse Embryonic Stem Cells

Matteo Perino, Guido van Mierlo, Chet Loh, Sandra M.T. Wardle, Dick W. Zijlmans, Hendrik Marks, and Gert Jan C. Veenstra

Supplemental Information

Two functional axes of positive feedback-enforced PRC2 recruitment in mouse embryonic stem cells

Matteo Perino*, Guido van Mierlo*, Chet Loh, Sandra M.T. Wardle, Dick Zijlmans, Hendrik Marks# & Gert Jan C. Veenstra#

Contents

- Supplemental Figures S1-S5
- Supplemental Tables: Legends corresponding to Table S1 and S2
- Supplemental Experimental Procedures
- Supplemental References

Figure S1

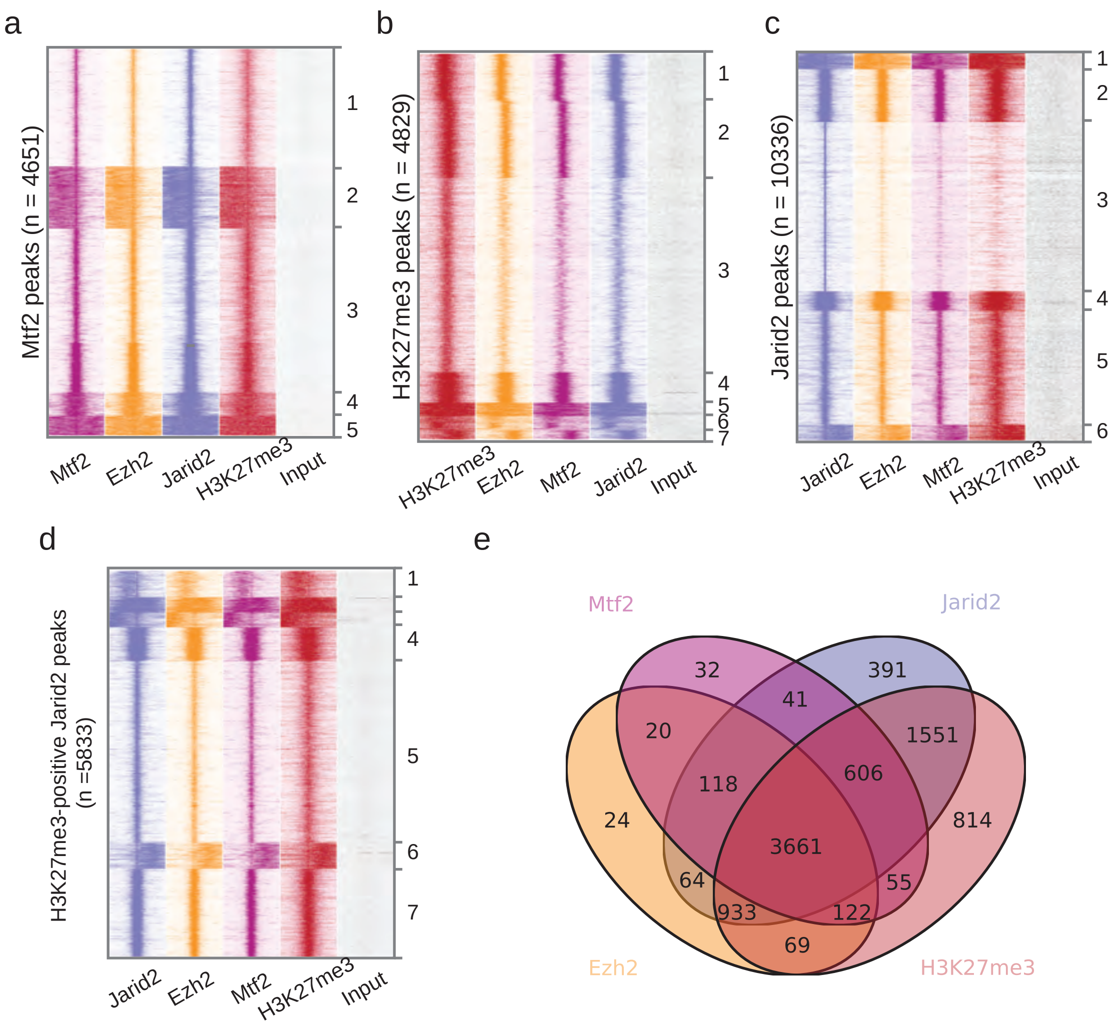

Supplementary Figure 1. a-d) Heatmap of WT ChIP-seq signal on the indicated peak set. H3K27me3-negative JARID2 peaks were excluded from further analysis. **e)** Venn diagram showing the overlap of peaks called for the ChIP-Seq of each protein independently. The ChIP-seq data represent two combined replicates from independent experiments.

Figure S2

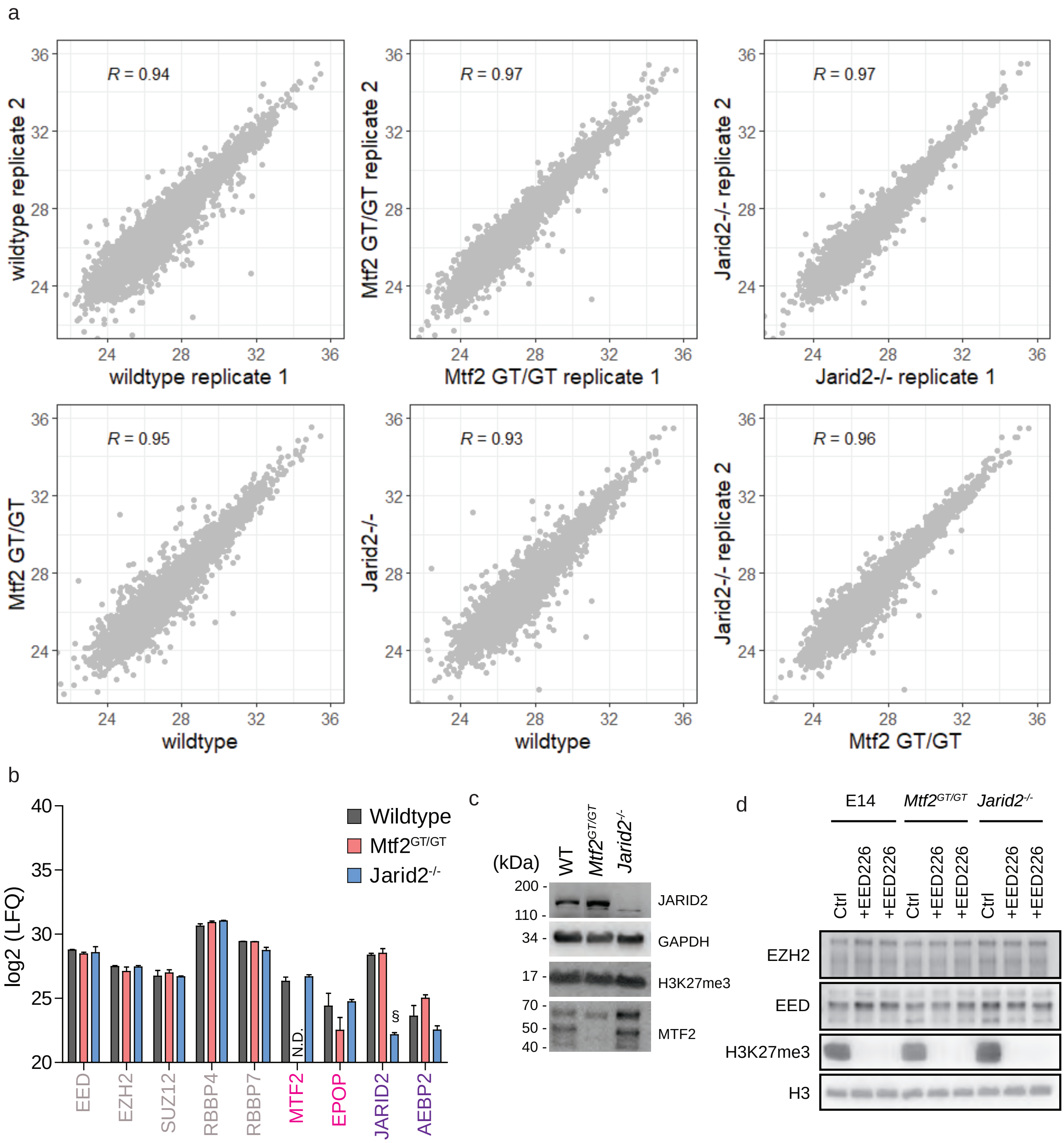

Supplementary Figure 2. a) Scatterplot of total proteome quantification across WT, Mtf2$^{GT/GT}$ and Jarid2$^{-/-}$ mESC. **b)** Quantification of the data shown in (a). § indicate detection of three residual JARID2 peptides in Jarid2$^{-/-}$. The data represent two combined replicates from independent experiments. **c)** Western blot validation of Mtf2 and Jarid2 mutant showing no residual protein. **d)** Western blot validation of H3K27me3 depletion in EED226 treated cells.

Figure S3

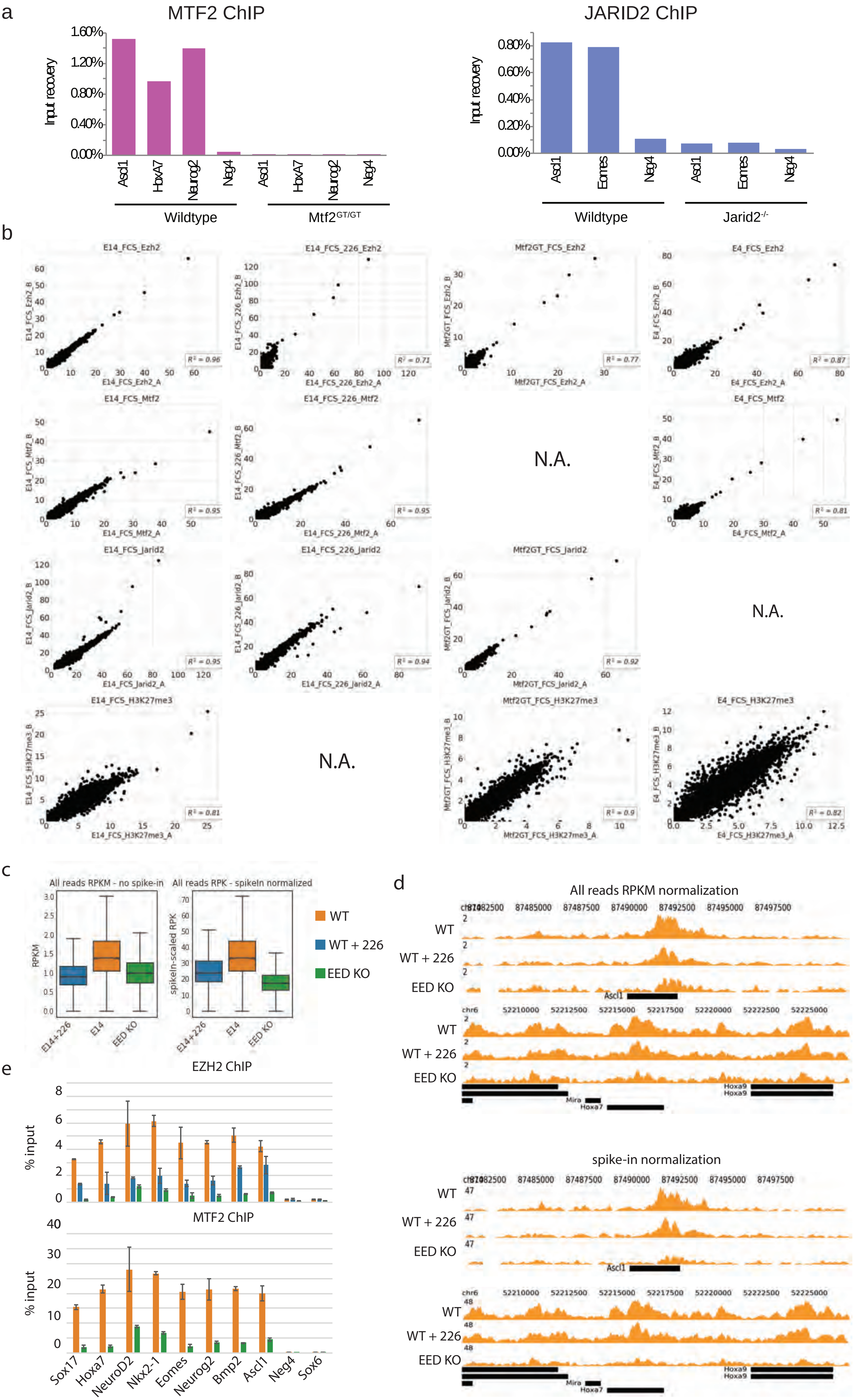
a
MTF2 ChIP
Input recovery
1.60%
1.20%
0.80%
0.40%
0.00%
Ascl1
HoxA7
Neurog2
Neg4
Wildtype
Mtf2GT/GT
JARID2 ChIP
0.80%
0.60%
0.40%
0.20%
0.00%
Eomes
Jarid2-/-
b
E14_FCS_Ezh2
E14_FCS_226_Ezh2
Mtf2GT_FCS_Ezh2
E4_FCS_Ezh2
E14_FCS_Mtf2
E14_FCS_226_Mtf2
N.A.
E4_FCS_Mtf2
E14_FCS_Jarid2
E14_FCS_226_Jarid2
Mtf2GT_FCS_Jarid2
E14_FCS_H3K27me3
Mtf2GT_FCS_H3K27me3
E4_FCS_H3K27me3
c
All reads RPKM - no spike-in
All reads RPK - spikeIn normalized
RPKM
spikeIn-scaled RPK
E14+226
E14
EED KO
WT
WT + 226
d
All reads RPKM normalization
spike-in normalization
Ascl1
Hoxa9
Mira
Hoxa7
e
EZH2 ChIP
MTF2 ChIP
% input
Sox17
Hoxa7
NeuroD2
Nkx2-1
Neurog2
Bmp2
Sox6

Supplementary Figure 3. a) qPCR validation of MTF2 and JARID2 mutants in the respective mutants. **b)** Scatterplot of peak RPKM showing high reproducibility of ChIP replicates. **c)** Peak intensity distribution of spike-in EZH2 ChIP-seq normalized with either RPKM or spike-in correction. Boxplots represent median and interquartile range (IQR; whiskers, 1.5 IQR). Outliers not shown. **d)** Examples of EZH2 ChIP-seq of the loci quantified in c). **e)** qPCR quantification of ChIP for EZH2 and MTF2 on multiple loci (n=2, independent experiments). Error bars represent standard deviation.

Figure S4

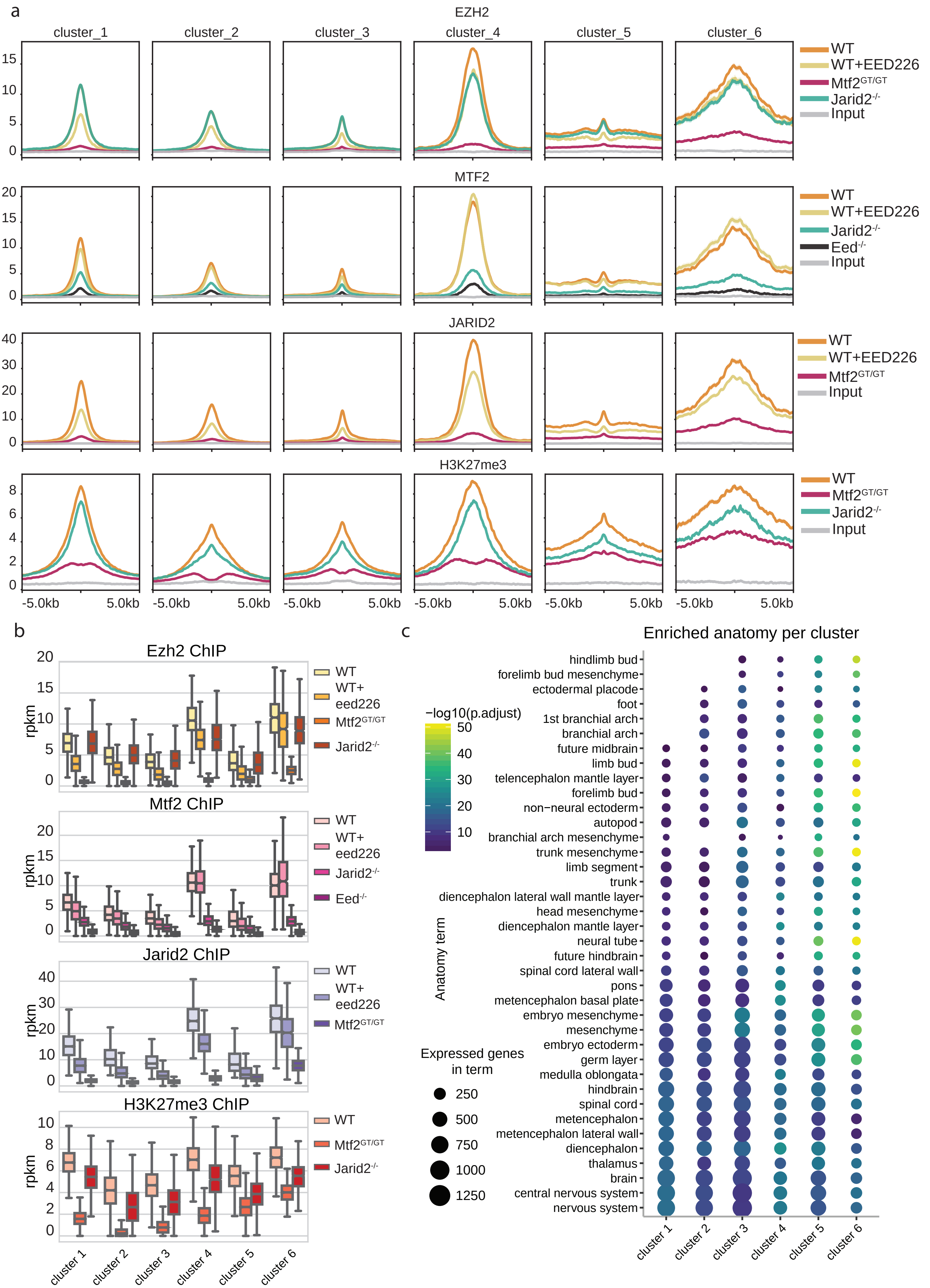
a
EZH2
cluster_1
cluster_2
cluster_3
cluster_4
cluster_5
cluster_6
WT
WT+EED226
Mtf2GT/GT
Jarid2-/-
Input
MTF2
Eed-/-
JARID2
H3K27me3
-5.0kb
5.0kb
b
Ezh2 ChIP
Mtf2 ChIP
Jarid2 ChIP
H3K27me3 ChIP
rpkm
WT+
eed226
cluster 1
cluster 2
cluster 3
cluster 4
cluster 5
cluster 6
c
Enriched anatomy per cluster
−log10(p.adjust)
Expressed genes
in term
Anatomy term
hindlimb bud
forelimb bud mesenchyme
ectodermal placode
foot
1st branchial arch
branchial arch
future midbrain
limb bud
telencephalon mantle layer
forelimb bud
non−neural ectoderm
autopod
branchial arch mesenchyme
trunk mesenchyme
limb segment
trunk
diencephalon lateral wall mantle layer
head mesenchyme
diencephalon mantle layer
neural tube
future hindbrain
spinal cord lateral wall
pons
metencephalon basal plate
embryo mesenchyme
mesenchyme
embryo ectoderm
germ layer
medulla oblongata
hindbrain
spinal cord
metencephalon
metencephalon lateral wall
diencephalon
thalamus
brain
central nervous system
nervous system

Supplementary Figure 4. **a)** Average plot of the ChIP signal shown in Fig 2a, centred on called peaks. **b)** Boxplot quantification of the signal shown in Fig. 2a. Boxplots represent the median and interquartile range (IQR; whiskers, 1.5 IQR). Outliers not shown. **c)** Enrichment of anatomical terms in the genes associated with peaks in the six clusters shown in Fig 2a. Enrichment over all genes. The ChIP-seq data represent two combined replicates from independent experiments.

Figure S5

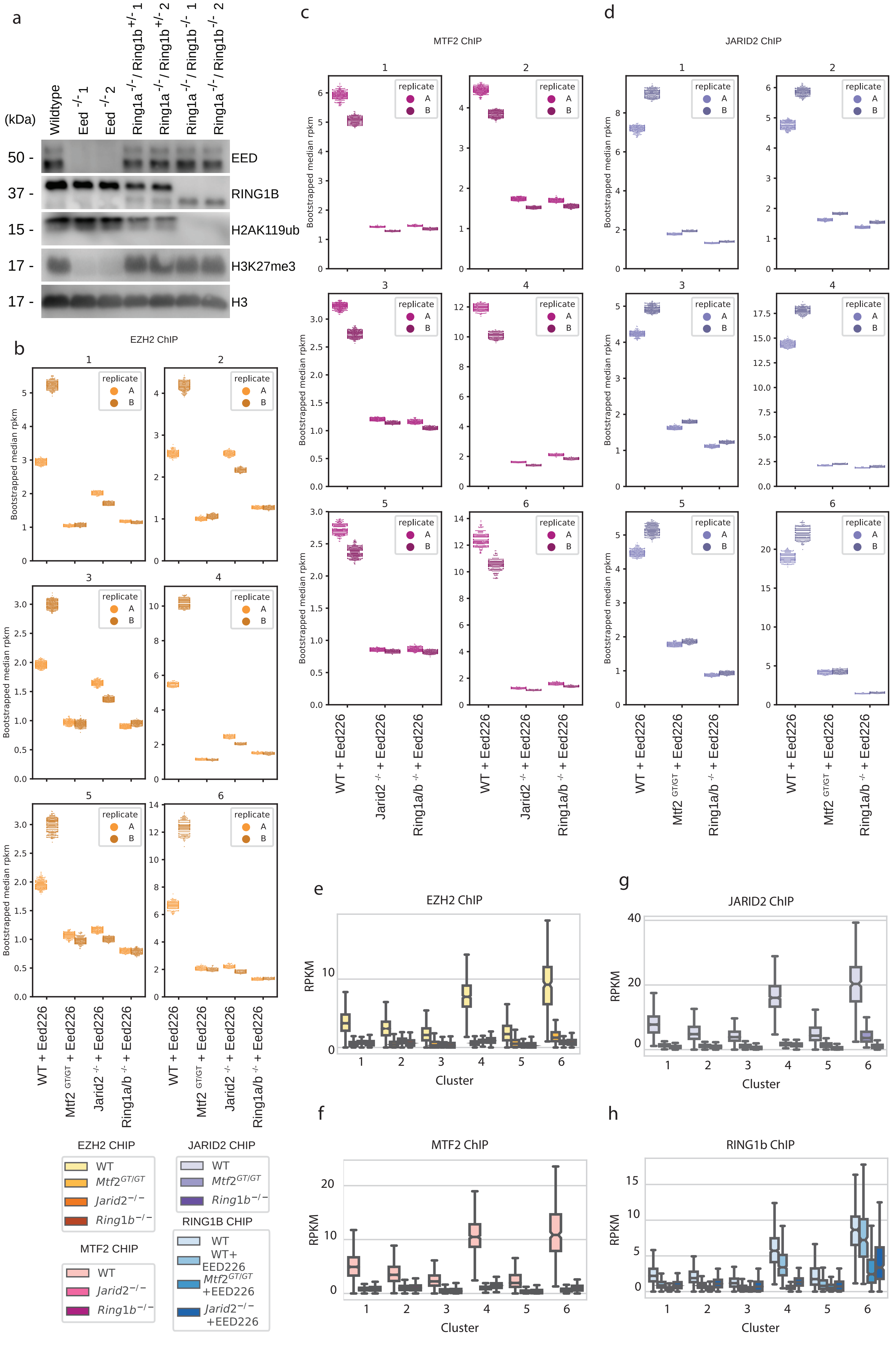
a
(kDa)
Wildtype
Eed -/- 1
Eed -/- 2
Ring1a -/- / Ring1b +/- 1
Ring1a -/- / Ring1b +/- 2
Ring1a -/- / Ring1b -/- 1
Ring1a -/- / Ring1b -/- 2
50 -
EED
37 -
RING1B
15 -
H2AK119ub
17 -
H3K27me3
17 -
H3
b
EZH2 ChIP
Bootstrapped median rpkm
replicate
A
B
WT + Eed226
Mtf2 GT/GT + Eed226
Jarid2 -/- + Eed226
Ring1a/b -/- + Eed226
c
MTF2 ChIP
d
JARID2 ChIP
e
EZH2 ChIP
RPKM
Cluster
f
MTF2 ChIP
g
JARID2 ChIP
h
RING1b ChIP
EZH2 CHIP
WT
Mtf2GT/GT
Jarid2−/−
Ring1b−/−
JARID2 CHIP
WT
Mtf2GT/GT
Ring1b−/−
MTF2 CHIP
WT
Jarid2−/−
Ring1b−/−
RING1B CHIP
WT
WT+ EED226
Mtf2GT/GT +EED226
Jarid2−/− +EED226

Supplementary Figure 5. a) Western blot validation of Eed$^{-/-}$ and Ring1ab$^{-/-}$ lines. **b-d)** Bootstrapping-based RPKM quantification (methods) of the signal in Fig 4 a-d. Each colored dot represents the median of one round of bootstrapping. Replicates are plotted independently. **e-h)** Boxplot quantification of data in Fig 4. Boxplots represent median and interquartile range (IQR; 763 whiskers, 1.5 IQR). Outliers not shown. Annotation of the colours is presented on the left of the boxplots. The data represent two combined replicates from independent experiments.

Supplemental Tables

Supplemental Table S1 and Table S2 are provided as Excel files and contain the following information:

- **Table S1:** Total proteome quantification
- **Table S2:** Chromatin proteome quantification (ChEP)

Supplemental experimental procedures

Embryonic stem cell culture

Eed-/- ESCs have been described by Schoeftner et al., 2006, Jarid2-/- ESCs have been described in Landeira et al. (Landeira et al., 2010). Mtf2 knockout (Mtf2GT/GT) (Li et al., 2011) and Ring1a-/- / Ring1b+/- ESCs (Endoh et al., 2008) were a kind gift from Haruhiko Koseki. Ring1b ESCs are knockout for Ring1a and trans-heterozygous for Ring1b (null/floxed). Full knockout of Ring1b was induced through treatment with Tamoxifen (OHT) for 2 days. All knockout ESCs were validated using western blot.

Western blot and antibodies

Cell pellets were dissolved in RIPA buffer at a density of 104 cells per µl and briefly sonicated to ensure proper cell lysis. Proteins denatured in SDS-PAGE gels were transferred onto PVDF membranes. Primary antibodies used were rabbit anti-MTF2 (ProteinTech; 16208-1-AP), rabbit anti-JARID2 (Novus Bio; NB100-2214), rabbit anti-H3K27me3 (Millipore; 07-449), rabbit anti-H3 (Abcam;1791). Secondary antibodies were HRP-conjugated anti-rabbit (Dako; P0217) and anti-mouse (Dako; P0161). Protein bands were visualized using Pierce ECL western blotting substrate (Thermo). Images were analyzed using ImageJ.

ChIP-sequencing

Cells were crosslinked in 1% PFA at room temperature for 8-10 min. The crosslinking reaction was quenched using 1.25M glycine and cells were harvested by scraping in buffer B (0.25% Triton X-100, 10 mM EDTA, 0.5 mM EGTA, 20 mM HEPES). The suspension was centrifuged for 5 min at 1600 rpm, 4 ˚C and the pellet was resuspended in 30 ml buffer C (150 mM, 1 mM EDTA, 0.5 mM EGTA, 50 mM HEPES) and rotated for 10 min at 4 ˚C. The nuclei were centrifuged 5 min at 1600 rpm, 4 ˚C and resuspended in incubation buffer (0.15% SDS, 1% Triton X-100, 150 mM NaCl, 1 mM EDTA, 0.5 mM EGTA, 20 mM HEPES) supplemented with Protease inhibitor. Nuclei were sonicated using a Biorupter Pico to obtain chromatin with an enriched DNA length of 300 bp. The chromatin was snap-frozen and stored at -80 ˚C until further use. For ChIP, sonicated chromatin was incubated overnight with the required antibody and pulled down using protein A/G magnetic beads (Perino et al., 2018). All ChIPs were performed using 3 µl/sample of the following antibodies: MTF2 (Aviva System Biology ARP34292, lot QC49692-42166), H3K27me3 (Millipore 07-449, lot 2717675), EZH2 (Diagenode C15410039, lot 003), JARID2 (Novus Biologicals NB100-2214, Lot E2), RING1B (Abcam, AB3832 lot GR86503-25) and spike in antibody (Active Motif 61686, lot 00419007).

For Spike-in ChIPs, 50ng of spike-in chromatin (*Drosophila melanogaster*) and a *Drosophila melanogaster*-specific H2Av antibody (Active Motif #61686, 2ug per chip) were added to selected chips. After washes, eluted chromatin was de-crosslinked overnight and purified with MinElute PCR Purification columns (Qiagen). After qPCR quality check for target enrichment, up to 5 ng/sample of ChIP was prepared for sequencing using the Kapa Hyper-prep Kit (Kapa Biosystems) using NEXTflex adapters (Bio Scientific), followed by 8-12 cycles amplification by PCR. After size-selection using E-gel (Invitrogen) or KAPA beads (Kapa Biosystems) to enrich for 300bp fragments, libraries were sequenced paired-end on an Illumina NextSeq500. qPCR analysis of ChIP DNA was performed with iQ SYBR Green Supermix (Bio-Rad) on a CFX96 Real-Time System C1000 Thermal Cycler (Bio-Rad). All the ChIP-Seq experiments in this study were performed at least in duplicate, from independent chromatin preparations.

ChIP-sequencing data analysis

Newly generated and previously published data sets (Perino et al., 2018) were processed in parallel with identical settings. To ensure maximum comparability (75bp single-end vs 42bp paired-end) and accurate quantification, all fastq files were trimmed to 42bp using fastx_trimmer (version 0.0.13.2), and in case of paired-end sequencing, only read_1 was used for analysis. All fastq files were mapped using bwa (version 0.7.10-r789), filtered to retain only uniquely mapping reads using mapping quality of 30 and samtools (version 1.7, flag -F 1024), then normalized for sequencing depth to produce bigwig. Peaks were called with MACS2-2.7 (Zhang et al., 2008) with qvalue 0.0001 using --call-summits for transcription factors and --broad for H3K27me3. Only peaks independently called in both replicates were used for downstream analysis. High-confidence peaks for each mark were obtained by merging peaks called in both replicates and overlapping by at least 50% of their length, and combined to obtain the list of all PRC2 peaks. Heatmaps of ChIP-Seq signals were generated using fluff v3.0.2 (Georgiou and van Heeringen, 2016) with the following settings. Bam files were used as input, the clustering method was set to k-means, the -r option was enabled for read-depth normalization (for non-spike in data). The heatmaps were clustered for dynamics using the "–g" option. Specifically, this -g option allows identifying dynamic patterns specifically at peak centers, for which a distance of 1kb in each direction from the peak center

was used in combination with Pearson correlation similarity as a distance metric. In contrast to the most common approach using Euclidian distance metrics across a wider area (typically +/- 5kb), this setting removes the influence of peak flanking regions on clustering, resulting in a clusters that reflect the peaks intensity at the summit rather than the general shape of the signal over several kb, thus better identifying dynamic changes across conditions at the most strongly bound region. As the peak flanking regions are ignored during clustering and the peak center considered as a single bin, information about the directionality of the signal outside the peak is not considered for clustering. ChIP metaplots were obtained with deeptools v 3.1.3 (Ramírez et al., 2016). Anatomy term enrichment was calculated using MouseMine (Motenko et al., 2015). RPKM bootstrapping analysis was performed using scipy (v 1.1.0). RPKM from the two independent ChIP-seq replicates were combined into a single pool. Values were drawn from this pool, recorded, and returned, such that every value could be drawn multiple times. For each bootstrapping round, a number of values matching the total number of PRC2 peaks was drawn, and the median plotted as one dot in the swarm plot. Confidence intervals (99.9%) were calculated from 100 bootstrapping events. DNA shape analysis was performed using the DNAshape package (Zhou et al., 2013).

ChIP-seq samples supplemented with *Drosophila* chromatin as spike-in were mapped on a combined mm10-dm6 genome, and reads mapping on multiple genomic locations or representing PCR duplicates were filtered out. The resulting bam files were split according to the species of origin and dm6 reads were used to calculate a per-sample scaling factor relative to the less deeply sequenced sample. ChIP-seq were first normalized based on the number of spike-in reads to obtain the number of reads per million of spike-in reads. This is achieved calculating a NormFactor with:

[1] NormFactor = 1e-6 * reads_ChIP_dm6

To account for the potentially varying starting amount of spike-in in different samples, we calculate F, the fraction of spike-in reads in input samples:

[2] F = Input_dm6 / (Input_dm6 + Input_mm10)

The final scaling factor S is calculated dividing the NormFactor of each sample by the F of its input:

[3] S = NormFactor / F

S is then scaled to obtain min(S) = 1.

[4] Scaled_S = S/min(S)

For comparison of peak intensity in RPKM and spike-in normalizations, reads per peak per kb (RPK) were calculated from the bam files and then normalized for either sequencing depth (RPKM) or spike-in scaling factor. This scaling factor was also used to down sample the bam files containing the mm10 reads used to produce the spike-in normalized heatmaps.

Chromatin Enrichment for Proteomics (ChEP)

Chromatin enrichment was performed as described previously, with minor adaptations (van Mierlo et al., 2019). In short, cells were crosslinked in 1% PFA for 10 minutes at 37 ˚C, washed twice in PBS, scraped and transferred to 2 ml tubes. Cells were resuspended in 1 ml ice-cold cell lysis buffer (25 mm TRIS pH 7.4, 0.1% Triton X-100, 85 mM KCl, 1X Roche protease inhibitor) and centrifuged at 2,300g for 5 min at 4 °C. The supernatant (cytoplasmic fraction) was removed and cell pellets were resuspended in 500 µl SDS buffer (10 mM TRIS pH 7.4, 10 mM EDTA, 4% SDS, 1X Roche protease inhibitor), incubated at RT for 10 minutes, topped up to 2 ml with Urea buffer (10 mM TRIS pH 7.4, 1 mM EDTA, 8 M urea) and centrifuged at 16,100g for 30 min at RT. The supernatant was discarded and this step repeated once. Next, the pellet was resuspended in 500 µl SDS buffer, topped up to 2 ml with SDS buffer and centrifuged at 16,100g for 30 min at RT. The cell pellet was taken up in storage buffer (10 mM TRIS pH 7.4, 1 mM EDTA, 25 mM NaCl, 10% glycerol, 1X Roche protease inhibitor) and sonicated in an NGS Bioruptor (Diagenode) to solubilize the pellet. The concentration of the resulting lysate was measured using a Qubit assay (Invitrogen). For sample preparation for mass spectrometry, 30 µg of protein extract was decrosslinked for 30 minutes at 95 ˚C by adding 4X decrosslinking buffer (250 mM Tris pH8.8, 2%SDS, 0.5M 2-mercaptoethanol) to final 1X. Decrosslinked chromatin extracts were processed and analyzed the same as whole-cell proteomes.

Supplementary references

Endoh, M., Endo, T.A., Endoh, T., Fujimura, Y., Ohara, O., Toyoda, T., Otte, A.P., Okano, M., Brockdorff, N., Vidal, M., et al. (2008). Polycomb group proteins Ring1A/B are functionally linked to the core transcriptional

regulatory circuitry to maintain ES cell identity. Development *135*, 1513–1524.

Georgiou, G., and van Heeringen, S.J. (2016). fluff: exploratory analysis and visualization of high-throughput sequencing data. PeerJ *4*, e2209.

Landeira, D., Sauer, S., Poot, R., Dvorkina, M., Mazzarella, L., Jørgensen, H.F., Pereira, C.F., Leleu, M., Piccolo, F.M., Spivakov, M., et al. (2010). Jarid2 is a PRC2 component in embryonic stem cells required for multi-lineage differentiation and recruitment of PRC1 and RNA Polymerase II to developmental regulators. Nat. Cell Biol. *12*, 618–624.

Li, X., Isono, K.-I., Yamada, D., Endo, T.A., Endoh, M., Shinga, J., Mizutani-Koseki, Y., Otte, A.P., Casanova, M., Kitamura, H., et al. (2011). Mammalian polycomb-like Pcl2/Mtf2 is a novel regulatory component of PRC2 that can differentially modulate polycomb activity both at the Hox gene cluster and at Cdkn2a genes. Mol. Cell. Biol. *31*, 351–364.

van Mierlo, G., Wester, R.A., and Marks, H. (2019). A Mass Spectrometry Survey of Chromatin-Associated Proteins in Pluripotency and Early Lineage Commitment. Proteomics *19*, e1900047.

Motenko, H., Neuhauser, S.B., O'Keefe, M., and Richardson, J.E. (2015). MouseMine: a new data warehouse for MGI. Mamm. Genome *26*, 325–330.

Perino, M., van Mierlo, G., Karemaker, I.D., van Genesen, S., Vermeulen, M., Marks, H., van Heeringen, S.J., and Veenstra, G.J.C. (2018). MTF2 recruits Polycomb Repressive Complex 2 by helical-shape-selective DNA binding. Nat. Genet. *50*, 1002–1010.

Ramírez, F., Ryan, D.P., Grüning, B., Bhardwaj, V., Kilpert, F., Richter, A.S., Heyne, S., Dündar, F., and Manke, T. (2016). deepTools2: a next generation web server for deep-sequencing data analysis. Nucleic Acids Res. *44*, W160–W165.

Schoeftner, S., Sengupta, A.K., Kubicek, S., Mechtler, K., Spahn, L., Koseki, H., Jenuwein, T., and Wutz, A. (2006). Recruitment of PRC1 function at the initiation of X inactivation independent of PRC2 and silencing. EMBO J. *25*, 3110–3122.

Zhang, Y., Liu, T., Meyer, C.A., Eeckhoute, J., Johnson, D.S., Bernstein, B.E., Nussbaum, C., Myers, R.M., Brown, M., Li, W., et al. (2008). Model-based Analysis of ChIP-Seq (MACS). Genome Biol. *9*, R137.

Zhou, T., Yang, L., Lu, Y., Dror, I., Dantas Machado, A.C., Ghane, T., Di Felice, R., and Rohs, R. (2013). DNAshape: a method for the high-throughput prediction of DNA structural features on a genomic scale. Nucleic Acids Res. *41*, W56–W62.
