# Supplementary material for: Two Functional Axes of Feedback-Enforced PRC2 Recruitment in Mouse Embryonic Stem Cells
Source: Stem Cell Reports. 2020 Aug 6;15(6):1287–300. doi: 10.1016/j.stemcr.2020.07.007 (PMC7724473; doi:10.1016/j.stemcr.2020.07.007)
Supplement: Document S2. Article plus Supplemental Information [file mmc4.pdf]

Stem Cell Reports
Article
OPEN ACCESS

Two Functional Axes of Feedback-Enforced PRC2 Recruitment in Mouse Embryonic Stem Cells

Matteo Perino,[1,3,5] Guido van Mierlo,[2,4,5] Chet Loh,[1] Sandra M.T. Wardle,[1] Dick W. Zijlmans,[2,4] Hendrik Marks,[2,6,*] and Gert Jan C. Veenstra[1,6,*]
[1]Department of Molecular Developmental Biology, Faculty of Science, Radboud Institute for Molecular Life Sciences, Radboud University, Nijmegen, the Netherlands
[2]Department of Molecular Biology, Faculty of Science, Radboud Institute for Molecular Life Sciences, Radboud University, Nijmegen, the Netherlands
[3]Presentaddress: Genome Biology Unit, European Molecular Biology Laboratory (EMBL), Heidelberg, Germany
[4]Presentaddress: Oncode Institute, Department of Molecular Biology, Radboud University, the Netherlands
[5]Co-first author
[6]Co-senior author
*Correspondence: h.marks@science.ru.nl (H.M.), g.veenstra@science.ru.nl (G.J.C.V.)
https://doi.org/10.1016/j.stemcr.2020.07.007

SUMMARY

Polycomb Repressive Complex 2 (PRC2) plays an essential role in gene repression during development, catalyzing H3 lysine 27 trimethylation (H3K27me3). MTF2 in the PRC2.1 sub-complex, and JARID2 in PRC2.2, are central in core PRC2 recruitment to target genes in mouse embryonic stem cells (mESCs). To investigate how PRC2.1 and PRC2.2 cooperate, we combined Polycomb mutant mESCs with chemical inhibition of binding to H3K27me3. We find that PRC2.1 and PRC2.2 mediate two distinct paths for recruitment, which are mutually reinforced. Whereas PRC2.1 recruitment is mediated by MTF2 binding to DNA, JARID2-containing PRC2.2 recruitment is more dependent on PRC1. Both recruitment axes are supported by core subunit EED binding to H3K27me3, but EED inhibition exhibits a more pronounced effect in *Jarid2* null cells. Finally, we show that PRC1 and PRC2 enhance reciprocal binding. Together, these data disentangle the interdependent interactions that are important for PRC2 recruitment.

INTRODUCTION

Cell fate specification during embryonic development requires tightly controlled epigenetic programs. A key component safeguarding these processes is Polycomb Repressive Complex 2 (PRC2), an enzymatic protein complex that catalyzes mono-, di-, and trimethylation of histone 3 lysine 27 (H3K27me1/2/3) and that plays an essential role in the establishment of cellular identity (Pengelly et al., 2013). The critical role of PRC2 during developmental processes is underscored by the embryonic lethality observed in mice lacking a functional PRC2 complex (Faust et al., 1998; O'Carroll et al., 2001; Pasini et al., 2007). PRC2 consists of the core subunits EED, SUZ12, and EZH2, the latter being the catalytic subunit. In addition, PRC2 contains multiple ancillary subunits exerting functions, such as guiding PRC2 to target genes and modulating its enzymatic activity. These include Polycomb-like proteins (PHF1, MTF2, or PHF19, also known as PCL1-3), EPOP (also known as C17ORF96), and PALI1/2 (also known as C10ORF12), which, together with the core subunits, form PRC2.1. Alternatively, the PRC2 core can associate with JARID2 and AEBP2 in another PRC2 sub-complex, referred to as PRC2.2 (Conway et al., 2018; van Mierlo et al., 2019a).

Within mouse embryonic stem cells (mESCs), the PRC2 core complex is mainly associated with MTF2 and EPOP (PRC2.1), or with AEBP2 and JARID2 (PRC2.2) (Kloet et al., 2016). Alternative PRC2.1 complexes containing either PHF1 or PHF19, and/or PALI1/2 are less abundant, in line with the very low expression of these proteins in mESCs (Kloet et al., 2016). In recent years, our understanding of Polycomb regulation in terms of recruitment and enzymatic activity has significantly increased. First, it has been shown that PRC2 can be recruited by the facultative subunits MTF2 and JARID2 in mESCs, while ablation of either EPOP or AEBP2 does not affect PRC2 localization (Beringer et al., 2016; Casanova et al., 2011; Grijzenhout et al., 2016; Landeira et al., 2010; Li et al., 2017; Liefke et al., 2016; Son et al., 2013). Second, after the first establishment of PRC2 binding, the complex can self-reinforce and spread from its target sites through an allosteric positive feedback loop by binding of the EED WD40 domain to H3K27me3 (Margueron et al., 2009; Poepsel et al., 2018). This mechanism is not sufficient for H3K27me3 maintenance during cell division (Laprell et al., 2017), thus underscoring the importance of continuous *de novo* recruitment of core PRC2 by its auxiliary subunits. Third, PRC2 can be recruited through variant PRC1, which binds to non-methylated DNA via its subunit KDM2B, and catalyzes the ubiquitination of H2A (H2AK119ub). This mark, in turn, can be bound by JARID2, resulting in PRC2.2 recruitment (Blackledge et al., 2020; Cooper et al., 2016; Kalb et al., 2014; Tamburri et al., 2020; Tavares et al., 2012). Finally, the H3K27me3 mark can be bound by canonical PRC1 via the CBX7 subunit, which contributes to gene repression by chromatin compaction (Blackledge et al., 2020; Isono et al., 2013;

This is an open access article under the CC BY license (http://creativecommons.org/licenses/by/4.0/).

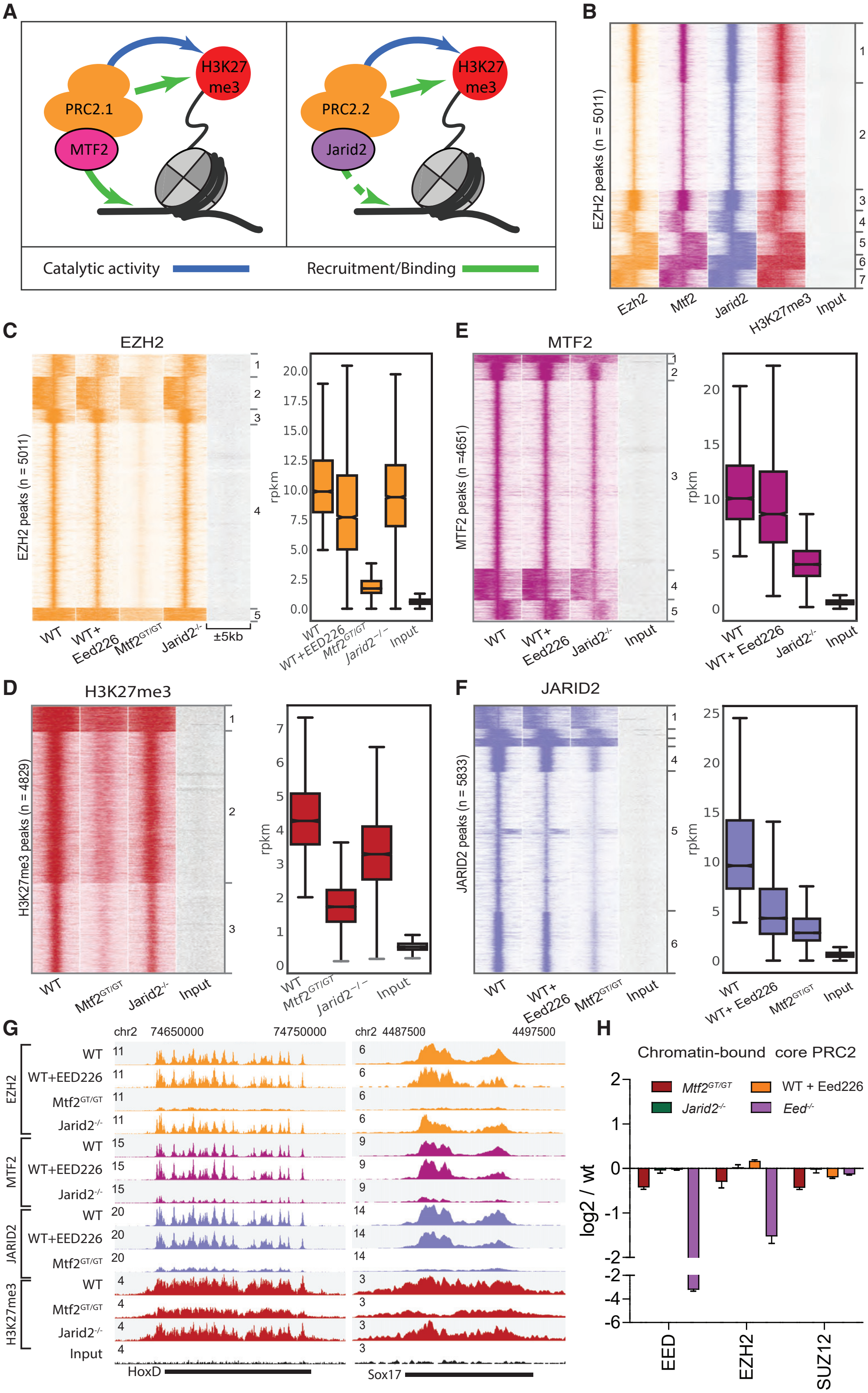

(legend on next page)

Lau et al., 2017; Morey et al., 2012; Tamburri et al., 2020). The bulk of H2A ubiquitination, however, is mediated by variant PRC1 complexes that contain one of the several PCGF proteins (Fursova et al., 2019).

It has become clear that MTF2 and JARID2 together are required for PRC2 recruitment to target genes in mESCs, as combined ablation of MTF2 and JARID2 in mESCs results in lack of PRC2 recruitment to target genes (Healy et al., 2019; Oksuz et al., 2018). This seems to depend to a large extent on MTF2-mediated DNA binding with a moderate contribution of JARID2 (Casanova et al., 2011; Li et al., 2017; Perino et al., 2018). Yet, while MTF2 and JARID2 are mutually exclusive within PRC2 complexes, the absence of either of the two partially reduces the binding of the other (Perino et al., 2018). This suggests that PRC2.1 and PRC2.2 could directly or indirectly synergize in establishing Polycomb at target genes. Whether such cooperativity exists, what the relative contribution of H3K27me3, PRC2.1, and PRC2.2 is, and how PRC1 plays a role in this process remains to be defined. Here, we combine a range of Polycomb mutant ESCs with chemical inhibition of PRC1 and PRC2 to address the complex interactions of the Polycomb system using chromatin immunoprecipitation sequencing (ChIP-seq). We assess the individual contributions of primary recruitment mechanisms established by JARID2, MTF2, and H3K27me3. Our data provide further evidence on the requirements of both PRC2.1 and PRC2.2 for PRC2 recruitment and H3K27 methylation (Healy et al., 2019; Højfeldt et al., 2019) but also elucidate the interdependent nature of their activity and how the EED-H3K27me3 interaction contributes to their recruitment. Our data indicate that H3K27me3-mediated recruitment of PRC2 can be compensated for by JARID2-mediated recruitment. Moreover, we provide evidence that this apparent redundancy is mediated through JARID2- and PRC1-deposited H2AK119ub. Together, our data support a model in which core PRC2 recruitment requires the concerted action of MTF2 and JARID2, as well as EED binding to H3K27me3. These modes of recruitment can be subdivided into two major axes, one that relies more on MTF2-mediated DNA binding, and the other depending to a larger extent on JARID2-PRC1- and H3K27me3-mediated recruitment. Moreover, these different recruitment axes appear to carry different weights across the genome. The data presented here demonstrate that the interactions between PRC2 sub-complexes are tuned depending on the genomic region and highlight their relevance in establishing PRC2 binding at target sites.

RESULTS

PRC2 Recruitment Mainly Depends on MTF2

Recent advances have pinpointed three main recruitment mechanisms of PRC2: (1) DNA-mediated recruitment via MTF2; (2) recruitment via JARID2; and (3) H3K27me3-mediated recruitment via EED (Figure 1A) (Cooper et al., 2016; Li et al., 2017; Margueron et al., 2009; Oksuz et al., 2018; Pasini et al., 2010; Perino et al., 2018). To investigate how they contribute to establishing PRC2 binding at target genes, we first evaluated whether these mechanisms act at the same genomic sites by performing chromatin immunoprecipitation followed by massive parallel sequencing (ChIP-seq) using antibodies against endogenous EZH2, H3K27me3, MTF2, and JARID2. We performed stringent peak calling (see Experimental Procedures) for EZH2 (n = 5,011 peaks) and determined the occupancy of H3K27me3, MTF2, and JARID2 on these peak sites, which revealed a near-perfect overlap (Figure 1B), as also shown previously (Healy et al., 2019; Højfeldt et al., 2019). The same result was obtained with peaks called for H3K27me3 or MTF2 (Figures S1A and S1B). By contrast, for JARID2 we observed a large number of sharp JARID2

Figure 1. Canonical PRC2 Recruitment Largely Relies on MTF2

(A) Schematic representation of the recruitment of PRC2.1 and PRC2.2. MTF2 binds to DNA, while the EED subunit of core PRC2 (orange) binds to H3K27me3 as part of an allosteric feedback loop. The EZH2 subunit of core PRC2 catalyzes H3K27 methylation. The PRC2.2 complex contains JARID2 but not MTF2. Both contain the core PRC2 subunits, but the interactions of the PRC2.1- and PRC2.2-specific subunits with chromatin are different. The arrow from JARID2 to DNA is dashed as DNA binding has been shown *in vitro* but not *in vivo* (Li et al., 2010).

(B) PRC2.1 (MTF2) and PRC2.2 (JARID2) co-localize to all EZH2 targets.

(C–F) Heatmap and RPKM quantification (boxplots) of PRC2 subunits and its catalytic product H3K27me3. EZH2 recruitment is heavily affected by the absence of MTF2, while absence of JARID2 and H3K27me3 has minor effects (C).The effect of MTF2 and JARID2 on EZH2 recruitment is reflected on H3K27me3 deposition (D). MTF2 is marginally affected by H3K27me3 removal, but its binding is reduced to approximately half the WT level in the absence of JARID2 (E). JARID2 recruitment is strongly reduced in the absence of either H3K27me3 or MTF2 (F). ChIP-seq profiles are highly reproducible (Figure S3B). Boxplots represent the median and interquartile range (IQR) (whiskers, 1.5 IQR). Outliers not shown.

(G) Genome browser examples of PRC2 binding to classical Polycomb targets.

(H) Proteomic quantification of chromatin-bound core PRC2 subunits. There is a visible, concordant decrease of bound PRC2 in the *Mtf2* mutant ESCs and no detectable changes in *Jarid2*$^{-/-}$.Error bars represent the SEM (n = 3 for WT and *Mtf2GT/GT*, n = 2 for WT+Eed226, *Jaric2*$^{-/-}$ and *Eed*$^{-/-}$).

See also Figures S1–S3. All ChIP-seq data represent two replicates from independent experiments.

peaks with little or no occupancy of the other PRC2 subunits (Figure S1C; cluster 3, n = 4,503 peaks) in addition to peaks shared with PRC2 and H3K27me3 (Figure S1D). This could indicate that JARID2 exerts functions independent of the PRC2 complex, as previously suggested in *Drosophila* (Herz et al., 2012). The Jarid2-only sites were excluded from consideration in this context and only the remaining, PRC2-positive peaks (Figures S1D and S1E) were used for subsequent analysis of PRC2 recruitment.

To understand how MTF2, JARID2, and H3K27me3 are involved in the recruitment of PRC2, we first focused on MTF2 and JARID2 and used knockout mESCs for these subunits (*Mtf2*$^{GT/GT}$ and *Jarid2*$^{-/-}$ cells, respectively). These mESCs lack MTF2 or JARID2, respectively, but globally retain wild-type (WT) levels of core PRC2 subunits in the context of a global proteome landscape similar to WT ESCs (Figures S2A–S2C; Table S1). We also confirmed that ChIP experiments for MTF2 in the *Mtf2*$^{GT/GT}$ ESCs and JARID2 in *Jarid2*$^{-/-}$ ESCs yielded no enrichment over negative loci, further validating the knockout ESCs as well as the antibodies (Figure S3A). ChIP-seq in these samples was highly reproducible (Figure S3B) and revealed a major reduction for EZH2 and H3K27me3 at target sites in *Mtf2* mutant cells, whereas the reduction in *Jarid2*$^{-/-}$ mESCs was milder (Figures 1C and 1D). These observations are in line with previous reports attributing a more prominent role for MTF2 in PRC2 recruitment in mESCs (Healy et al., 2019; Højfeldt et al., 2019; Li et al., 2017; Oksuz et al., 2018; Perino et al., 2018). To investigate whether PRC2.1 and PRC2.2 mediate recruitment of each other, we analyzed the genomic locations bound by MTF2 and JARID2 in the knockout cells. This revealed that MTF2 and JARID2 mutually affect each other's recruitment (Figures 1E and 1F). To investigate the role of the allosteric EED feedback loop, we extended our analysis to WT mESCs treated with the chemical inhibitor EED226. By binding the EED WD40 domain, EED226 interferes with the binding of EED to H3K27me3 while simultaneously inducing a conformational change that impedes stimulation of the EZH2 catalytic activity by EED (Qi et al., 2017). EED226 does not disturb physical associations between core PRC2 subunits, or their expression level (Qi et al., 2017). We first confirmed that EED226 treatment removed H3K27me3, validating its efficacy, without affecting core PRC2 levels (Figure S2D). Next, we performed ChIP-seq for EZH2, MTF2, and JARID2. This revealed that EED226 treatment resulted in a reduced recruitment of EZH2, MTF2, and JARID2 (respectively, 77%, 85%, and 41%; Figures1C–1F). This indicates that JARID2 binding depends more strongly on H3K27me3. Thus, the reduction of H3K27me3 in *Mtf2* mutant cells could largely explain the reduction of JARID2 binding in this cell line. By contrast, MTF2 recruitment is hardly affected by EED inhibition (Figure 1E), therefore the effect of JARID2 on MTF2 binding might rely on a direct or indirect stabilization of PRC2.1 on chromatin. Finally, we checked PRC2 binding by proteomic analysis of chromatin-bound proteins, which recapitulated our ChIP-seq findings. Despite ChIP peaks representing a minor fraction of the genome, and PRC2 having been reported to bind outside canonical targets to deposit H3K27me2 and H3K27me3 genome-wide (Ferrari et al., 2014; van Mierlo et al., 2019b), we identify a consistent reduction of core PRC2 subunits in bulk chromatin of *Mtf2* mutant cells (Figures 1H; Table S2), supporting the role of MTF2 in PRC2 recruitment. We did not observe reductions of core PRC2 on total chromatin in *Jarid2*$^{-/-}$- or EED226-treated ESCs, likely owing to less pronounced effects of these perturbations on PRC2 recruitment, in line with previous observations (Healy et al., 2019). To further validate our quantifications, we repeated a subset of the EZH2 ChIP-seq experiments, including *Drosophila* chromatin as spike-in for normalization and complemented it with ChIP-qPCR quantifications. This revealed good concordance between spike-in normalization and genome-wide reads per kilobase of peak per million mapped reads (RPKM)-based normalization for the same samples (Figures S3C–S3E). Taken together, these data corroborate previous observations regarding the prominent role of MTF2 in the recruitment of PRC2 and H3K27 methylation (Healy et al., 2019; Højfeldt et al., 2019; Perino et al., 2018). Moreover, the data show that PRC2.1 and PRC2.2 depend to a different extent on EED binding to H3K27me3.

Stratification of Polycomb Binding Reveals Two Major Types of Binding Sites

We noticed that several of the clusters observed in Figure 1 showed distinct characteristics, such as the strength of binding or the width of the peaks (heatmaps in Figures 1C–1F). To uncover the quantitative heterogeneity of PRC2 target sites in response to various perturbations, we used k means clustering and determined the optimal number of clusters to be six (elbow method). Also, recent work showed that the recruitment of MTF2 to some sites is lost completely in the absence of PRC2, whereas residual binding is observed at other loci (Perino et al., 2018), suggesting that distinct modes of recruitment guide PRC2 to different genomic regions. To determine if and how PRC2 recruitment might differ among genomic loci, we included in our analysis MTF2 ChIP-seq data of mESCs lacking EED (Perino et al., 2018), a condition with strongly reduced PRC2 core protein expression and binding (Figure 1H) (Højfeldt et al., 2018). We also included BioCap data (Long et al., 2013) to identify regions free of DNA methylation that can be bound by MTF2 (Perino et al., 2018), and H3K4me3 ChIP-seq data of WT mESCs (Perino et al., 2018) to identify bivalent promoter elements that comprise the

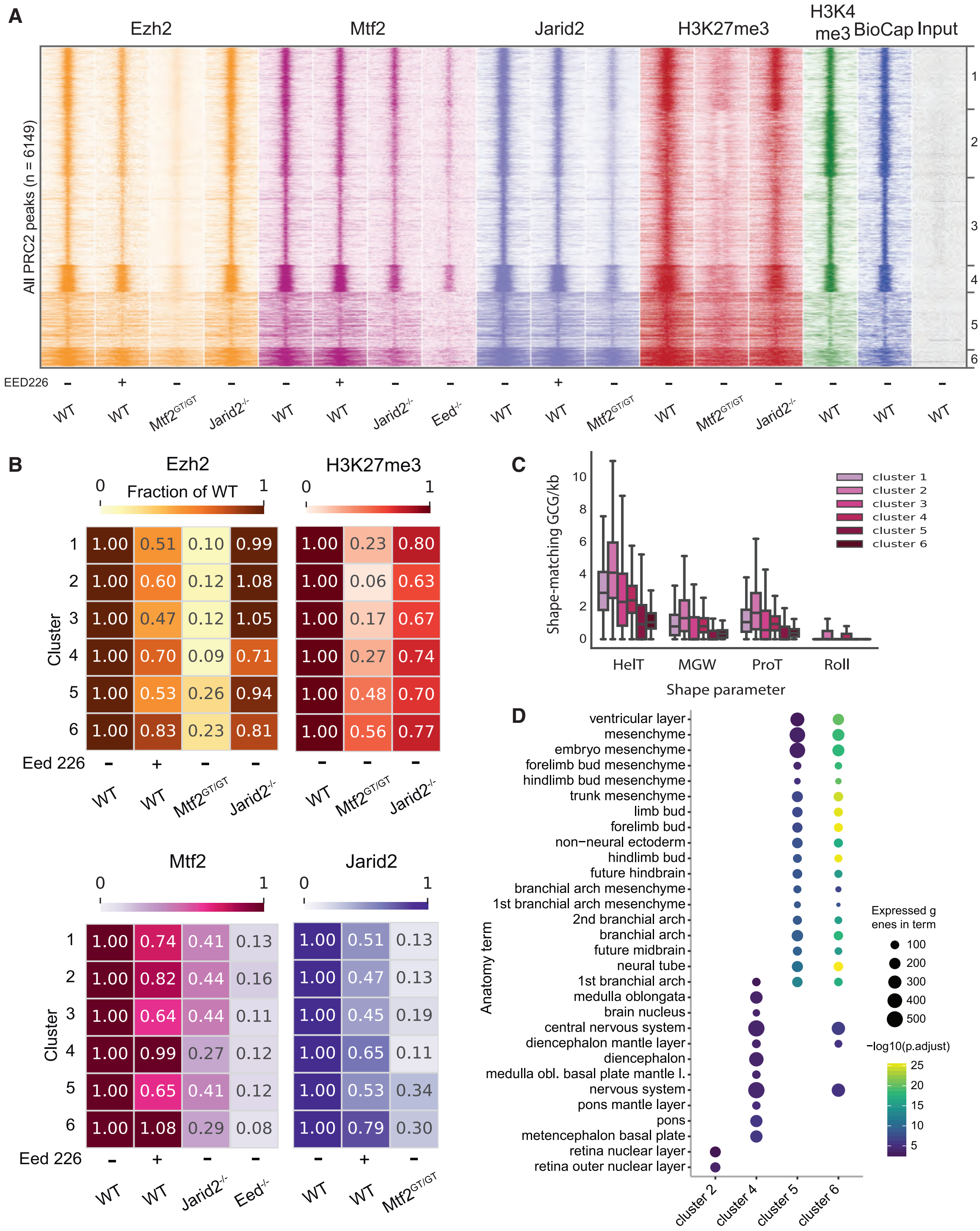

Figure 2. Identification of Two Distinct Classes of Polycomb Target Regions, Which Rely on Different Mechanisms of PRC2 Recruitment

(A) Clustering of all PRC2 targets using ChIP-seq data in multiple PRC2 mutants. Clusters 1–4 are unmethylated CpG islands (strong BioCap) signal, showing bivalent marks in WT (H3K4me3 and H3K27me3). These regions display a heavy reduction of EZH2 recruitment in the MTF2 mutant, milder effects of H3K27me3 absence (EED226 treatment), and little or no effect of JARID2 absence. The intensity of MTF2 binding depends on both H3K27me3 and JARID2 but binding is still clearly detectable even in the absence of PRC2 core (*Eed*$^{-/-}$). This indicates primary binding of MTF2 to DNA, reinforced by other mechanisms, such as JARID2-mediated recruitment, which in turn also depends on

(legend continued on next page)

majority of Polycomb targets in mESCs (Brookes et al., 2012). We combined these data with those shown in Figure 1 and clustered them on the common set of PRC2-bound regions (n = 6,149 peaks). To identify dynamic patterns specifically at peaks, we clustered reads close to the peak center (±1 kb) using Pearson correlation as a distance metric, which revealed six major clusters (Figure 2A; cluster 1, n = 1,215; cluster 2, n = 1,285; cluster 3, n = 1,686; cluster 4, n = 529; cluster 5, n = 1,073; cluster 6, n = 361). Clusters 1–4 display strong and sharply localized PRC2 binding and H3K27me3 deposition in WT conditions, accompanied by BioCap and H3K4me3 signals, thus displaying a signature resembling that of bivalent promoters (Bernstein et al., 2006). Clusters 5–6 instead show more dispersed binding, wider H3K27me3 domains, relatively low BioCap signal (indicating the absence of unmethylated CpG islands), and weaker H3K4me3 signals (fewer active or poised promoters). We observed that the consequences of the perturbations varied per cluster (Figures 2B, S4A, and S4B). The H3K27me3 signal, for example, is affected more in clusters 1–4 (reduced to 6%–27%) compared with clusters 5–6 (48%–56%) in *Mtf2*$^{GT/GT}$ ESCs (Figures 2B, top right, S4A, and S4B). Similar patterns are observed for EZH2 (Figure 2B, top left; 9%–12% versus 23%–26%) and JARID2 recruitment (Figure 2B, bottom right; 11%–19% versus 30%–34%). These observations further corroborate recent observations that narrow PRC2 target sites (here clusters 1–4) are more dependent on PRC2.1-mediated recruitment (Healy et al., 2019). We recently found that MTF2 binding to unmethylated CpGs is associated with a specific shape of the DNA, characterized by a reduced helix twist (Perino et al., 2018). Therefore, we performed *in silico* prediction of the DNA shape characteristics of the genomic sequences in each cluster. This revealed that shape-matching GCG trinucleotides previously shown to recruit MTF2 (Perino et al., 2018) are much more prevalent in clusters 1–4 (Figure 2C), providing a potential explanation for the higher dependence on MTF2 in these clusters. Recent findings showed that the affinity of PCL-containing PRC2 is strongly increased by dimerization on target DNA (Chen et al., 2020). As the absence of EED results in the lack of assembled PRC2 core and, therefore, of PRC2.1 dimerization, this could suggest that clusters 1–4, in contrast to clusters 5–6, contain sufficient MTF2 motifs for it to bind its targets also without dimerization-induced stability, albeit at lower levels than in WT cells. As previous reports suggested that Polycomb target sites contain distinct gene sets (Brookes et al., 2012), we tested whether clusters 1–4 and 5–6 were also enriched for different sets of genes. When compared with all the mouse genes, we observed that all clusters are enriched for genes associated with the development of body structures (Figure S4C), as is characteristic for Polycomb target genes (Brookes et al., 2012). When stratifying the clusters by enrichment over PRC2-targeted genes instead of all genes, we observed that clusters 5 and 6 are strongly enriched for genes related to body plan formation, including limb bud, trunk, and branchial arches mesenchyme (Figure 2D), while clusters 2 and 4 show a stronger enrichment for neural structures (Figure 2D) and clusters 1 and 3 show no specific enrichment. In addition, all Hox genes, which are highly conserved master regulators of embryonic development and body plan specification, are exclusively present in clusters 5–6. Collectively, these analyses further support the existence of two distinct classes of Polycomb target regions associated with distinct sets of developmental genes.

Pronounced Loss of PRC2 Binding by EED Inhibition in Jarid2 Null Cells

Our analyses allowed us to investigate the individual contributions of MTF2, JARID2, and H3K27me3 for PRC2 recruitment. However, the ablation of individual interactions does not reveal the extent to which they compensate for each other. Specifically, we wondered to what extent the EED-H3K27me3 interaction is redundant with MTF2 and JARID2. Thus, we combined knockouts of MTF2 and JARID2 with inhibition of H3K27 methylation binding by EED using EED226 treatment. Treatment of *Mtf2*$^{GT/GT}$ ESCs with EED226 would only leave JARID2-mediated recruitment intact, while combined removal of JARID2 with EED226 treatment would leave only the contribution of MTF2-mediated recruitment (cf. Figure 1A). In both situations, treatment with EED226 resulted in the bulk removal of H3K27me3 without affecting the levels of core

both H3K27me3 and MTF2. Clusters 5 and 6 have lower BioCap and H3K4me3 signals and, while still affected by the absence of MTF2, this has a much less marked effect on the recruitment of both EZH2 and JARID2, and on H3K27me3 deposition.

(B) WT-normalized, input-subtracted RPKM quantification of the signal shown in (A).

(C) Quantification of GCG trinucleotides matching DNA shape requirement for MTF recruitments as defined in Perino et al. (2018). Clusters 1–4 are strongly enriched in shape-matching GCGs, indicating the potential for strong DNA-mediated MTF2 recruitment. Boxplots represent the median and interquartile range (IQR) (whiskers, 1.5 IQR).

(D) Enrichment of anatomical terms in the genes associated with peaks in the six clusters. Enrichment within PRC2 targets. Cluster 4 shows strong enrichment for CNS structures, clusters 5 and 6 for limb and branchial arches tissues and mesenchyme. See Figure S4C for the full overview. Note that clusters 1 and 3 are missing as these did not display significantly enriched gene ontology terms. See also Figure S4. The ChIP-seq data represent two replicates from independent experiments.

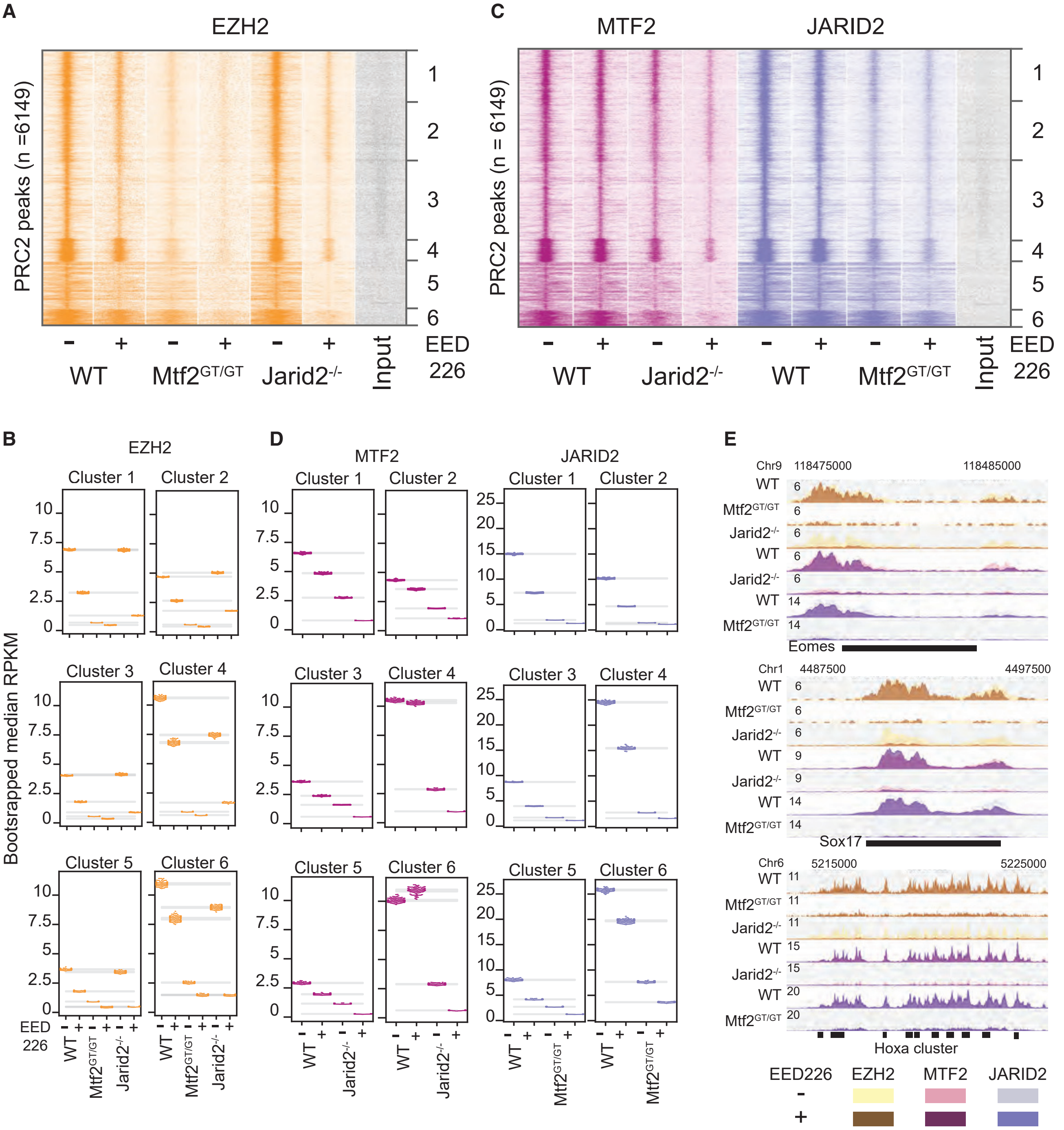

Figure 3. The H3K27me3 Feedback Loop and JARID2 Are Mutual Backups for PRC2 Recruitment

(A) Heatmap showing the cluster-specific effect of H3K27me3 depletion on the binding of EZH2. WT and *MTF2$^{GT/GT}$* show a mild reduction of EZH2 binding when treated with the EED226 inhibitor, while the treatment is highly synergistic with the depletion of JARID2.

(B) Bootstrapping-based RPKM quantification (methods) of the signal in (A). Each colored dot represents the median of one round of bootstrapping, gray bars represent 99.9% confidence interval for the mean of bootstrapped values in each condition and cluster.

(C) Treatment with EED226 further affected MTF2 recruitment in *Jarid2$^{-/-}$* and JARID2 recruitment in *Mtf2$^{GT/GT}$*, with the former leading to a recruitment pattern closely resembling the *Eed$^{-/-}$* line (cf. Figure 2A), highlighting the recruitment differences between clusters 1–4 and 5–6.

(legend continued on next page)

PRC2 subunits EZH2 and EED (Figure S2D). We examined the effect on core PRC2 recruitment to target genes by performing ChIP-sequencing of EZH2 in *Mtf2*$^{GT/GT}$ + EED226 ESCs and *Jarid2*$^{-/-}$ + EED226 mESCs. Inspection of the EZH2 signal revealed a slight decrease of EZH2 recruitment in *Mtf2*$^{GT/GT}$ + EED226 mESCs, compared with the already severe phenotype caused by MTF2 depletion alone (Figures 3A, 3B, 3E). Interestingly, although at most target locations the absence of JARID2 or treatment with EED226 alone had only a moderate effect on PRC2 recruitment, their combination resulted in a dramatic decrease of EZH2 recruitment (Figures 3A, 3B, 3E). This could suggest that JARID2 and H3K27me3 are redundant for PRC2 recruitment or can compensate for each other. Besides, this demonstrates that MTF2-mediated recruitment, by itself, is not sufficient to establish full core PRC2 recruitment, but requires PRC2.2 and the EED-mediated positive feedback loop.

We extended our analyses by performing ChIP-seq for JARID2 in *Mtf2*$^{GT/GT}$ + EED226 mESCs and MTF2 in *Jarid2*$^{-/-}$ + EED226 mESCs. Removal of both JARID2 and H3K27me3 further reduced MTF2 recruitment, and especially in clusters 5–6, MTF2 recruitment was near-zero (Figures 3C–3E). This shows that MTF2, and hence PRC2.1, are recruited to these broad Polycomb domains through PRC2.2 and the EED-positive feedback loop. This is in agreement with the strongly attenuated MTF2 binding in *Eed*$^{-/-}$ mESCs (Figures 2A and 2B) and the absence of enrichment for GCG trinucleotides compatible with MTF2 binding (Figures 2A–2C). JARID2 binding in *Mtf2*$^{GT/GT}$ + EED226 ESCs was reduced in all clusters, but a marginally stronger reduction was observed in clusters 5–6 (Figures 3C and 3D). Together, these data uncover the contribution of the EED-H3K27me3 interaction to PRC2 recruitment, in particular for PRC2.2, and show that the relative importance of PRC2.1 and PRC2.2 differs across the genome.

JARID2 Recruitment Is Largely Dependent on PRC1

Recent observations have indicated that JARID2 can be recruited through binding to H2AK119ub deposited by variant PRC1 (vPRC1) (Blackledge et al., 2014, 2020; Cooper et al., 2014; Tamburri et al., 2020). Our analyses indicated the importance of EED binding to H3K27me3 when JARID2 is absent. Therefore, we hypothesized that cells in which both EED binding is inhibited and H2AK119ub is simultaneously absent might phenocopy *Jarid2*$^{-/-}$ + EED226 mESCs. To test this, we used *Ring1a/b* double-mutant mESCs treated with EED226 (*Ring1a/b*$^{-/-}$ + EED226) and performed ChIP-seq of EZH2, MTF2, and JARID2 in these ESCs, after additional validation of the knockout lines (Figure S5A). Interestingly, we observed that the EZH2 and MTF2 profiles obtained in *Jarid2*$^{-/-}$ + EED226 and *Ring1a/b*$^{-/-}$ + EED226 were almost indistinguishable (Figures 4A–4C, light and dark blue lines in Figures 4B and S5B–S5F). In addition, JARID2 binding was affected in *Ring1a/b*$^{-/-}$ + EED226 cells (Figures 4D, S5D, and S5G) to a larger extent than with EED226 treatment alone, suggesting that these mechanisms are additive. This suggests that JARID2 and vPRC1 together recruit PRC2.2. Also, EZH2 and MTF2 recruitment was nearly abolished in broad peaks (clusters 5–6) but still retained, although at low levels, in narrow peaks (clusters 1–4), which is in line with recent observations highlighting a more prominent role for vPRC1 in PRC2 recruitment to broad H3K27me3 regions (Healy et al., 2019). We noted that low residual JARID2 recruitment is retained when the absence of H2AK119ub deposition and EED binding to H3K27me3 are combined (*Ring1a/b*$^{-/-}$ + EED226 condition). This suggests that additional mechanisms mediate low levels of JARID2 recruitment, for example, through binding of JARID2 to DNA (Li et al., 2010) or RNA (Brockdorff, 2013; Kaneko et al., 2014). To extend our analysis on PRC1-PRC2 interdependencies and disentangle the roles of H3K27me3 versus PRC2 subunits in PRC1 recruitment, we performed RING1B ChIP-seq in WT, *Mtf2*$^{GT/GT}$, and *Jarid2*$^{-/-}$ in the presence of EED226. While inhibiting EED binding to H3K27me3 in WT ESCs had a limited effect on RING1B recruitment (Figures 4E–4G and S5H), the combination with the absence of either MTF2 or JARID2 results in a stronger reduction of RING1B binding (Figures 4E–4G). While the Polycomb dogma posits that PRC1 and PRC2 do not physically interact and mutually affect each other only via their catalytic products, these data might suggest that PRC2 also contributes to PRC1 recruitment independently of H3K27me3. For example, it is conceivable that the physical presence of PRC2 at target genes (which is strongly reduced in *Mtf2*$^{GT/GT}$ + EED226 and *Jarid2*$^{-/-}$ + EED226 ESCs) stabilizes PRC1 binding to chromatin, for example, by stabilizing KDM2B binding (Oksuz et al., 2018).

DISCUSSION

The mechanisms that guide and maintain PRC2 at target sites have been the focus of extensive research, yet have long remained enigmatic. Although the allosteric feedback loop mediated by EED is important for the spreading of

(D) Bootstrapping-based RPKM quantification (methods) of the signal in (C) similar as in (B).
(E) Genome browser view of example Polycomb targets. For each genotype two tracks are overlaid: the darker colors represent EED226-treated samples, the lighter color untreated cells. The ChIP-seq data represent two replicates from independent experiments.

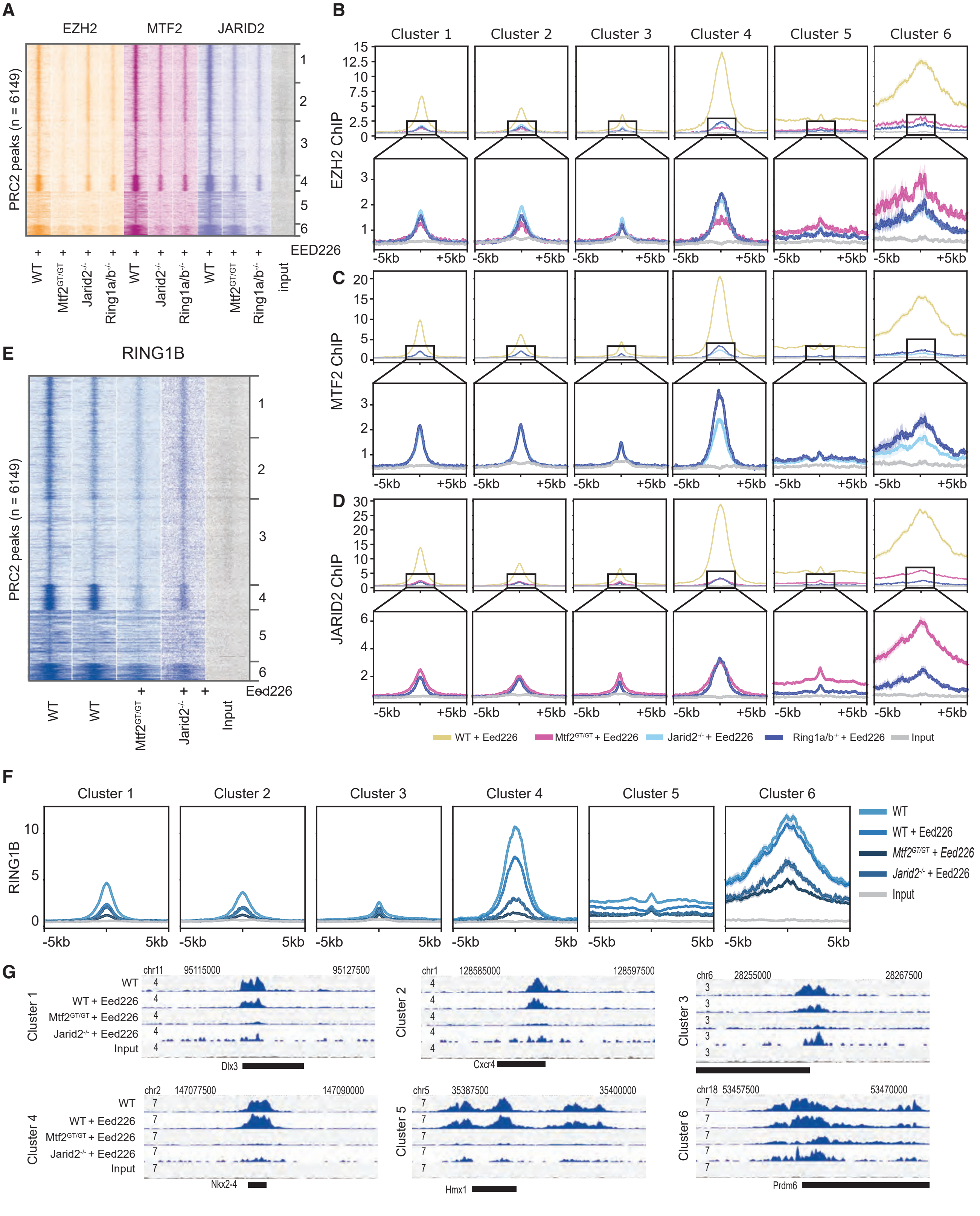

(legend on next page)

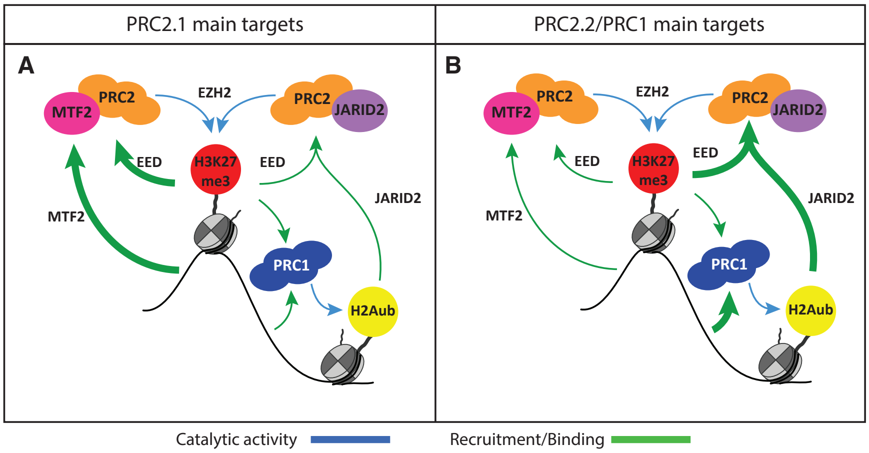

Figure 5. Model of PRC2 Recruitment Mechanisms and Interactions

(A) On PRC2.1 main targets (clusters 1–4) relatively little MTF2 binding is sufficient to kickstart the EED-positive feedback loop which heavily relies on JARID2. As primary recruitment is mediated to a large extent via MTF2, such a loop can still exist in the absence JARID2. In the absence of H3K27me3, an alternative route can take over that requires JARID2 binding to H2AK119ub.

(B) On PRC2.2/PRC1 targets (clusters 5–6), instead, Polycomb binding is initiated by PRC1 that, upon H2AK119ub deposition, is followed by JARID2-containing PRC2.2. These regions also see the presence of MTF2 in physiological conditions, but this is the result of indirect recruitment via the PRC2 core binding to PRC2.2-initiated H3K27me3 deposition.

PRC2 away from its initial nucleation site (Margueron et al., 2009), the mere presence of H3K27me3 is not sufficient to maintain PRC2 at its target genes (Laprell et al., 2017). This indicates that continuous DNA-mediated and target-specific recruitment or stabilization is required to attract PRC2 to newly replicated chromatin fibers (Laprell et al., 2017). The recent discoveries of facultative PRC2 subunits and the presence of functionally distinct sub-complexes have greatly advanced our understanding of PRC2 recruitment and maintenance (Hauri et al., 2016; Smits et al., 2013). In particular, individual ablation of all prime facultative subunits in mESCs revealed a major role for MTF2 in PRC2 recruitment, which, together with JARID2, mediates the initial PRC2 binding to the initiation sites ("nucleation sites") (Li et al., 2017, 2010; Oksuz et al., 2018; Perino et al., 2018).

In this study, we dissect the relative contributions of various recruitment mechanisms and the extent to which they are interdependent. At face value, it appeared that JARID2 contributes less to core PRC2 recruitment compared with MTF2, as *Jarid2* null cells displayed only a moderate reduction in EZH2 binding. However, we found that inhibition of EED uncovered a profound contribution of JARID2 to overall PRC2 recruitment. In addition, the experiments revealed a significant interdependence of PRC2.1 and PRC2.2. In part, reduced PRC2.2 recruitment in *Mtf2* null cells can be explained by reduced levels of H3K27me3, whereas PRC2.1 binding may not only be affected by the binding of EED to H3K27me3, but also by other mechanisms, such as EED binding to methylated JARID2 (Sanulli et al., 2015). Our observations underscore the importance of MTF2 for a significant proportion of PRC2 recruitment. Although mESCs also display a low expression of the other PCL proteins, PHF1 and PHF19, these are hardly detectable via mass spectrometry approaches (whole-cell proteomes and chromatin-associated proteomes, cf. Figures 1H and S2). These proteins are also not able to compensate for the loss of MTF2, at least in mESCs, as mESCs lacking all three PCL proteins display similar PRC2 recruitment to *Mtf2* knockout mESCs (Healy et al., 2019; Højfeldt et al., 2019). While PHF1 and PHF19 might not play a dominant role in PRC2.1 recruitment in ESCs, this might change upon differentiation of ESCs during which the

Figure 4. JARID2 Recruitment Is Largely Dependent on PRC1

(A) Heatmap showing EZH2, MTF2, and JARID2 binding in the absence of H3K27me3 in PRC2 and PRC1 mutant lines. In the absence of H3K27me3, JARID2, and RING1A/B mutant phenocopy each other with respect to EZH2 and MTF2 binding, suggesting that JARID2 and RING1B act along the same PRC2 recruitment axis. JARID2 recruitment is also strongly affected by the absence of RING1A/B, in line with the JARID2-mediated PRC2 recruitment via binding to PRC1-deposited H2AK119ub.

(B–D) Average plot of the ChIP signal shown in (A), for EZH2 (B), MTF2 (C), and JARID2 (D) centered on called peaks. Lower panels represent the same data with cropped y axis, for better visualization.

(E) Heatmap showing RING1B binding in the discussed conditions. RING1B is only mildly affected by removing H3K27me3 using EED226 (~40%). Binding is further attenuated in MTF2 and JARID2 mutant ESCs.

(F) Average plot of the ChIP signal shown in (E), centered on called peaks.

(G) Examples of loci of the data as shown in (E). See also Figure S5. The ChIP-seq data represent two replicates from independent experiments.

stoichiometry of MTF2 is strongly reduced and that of PHF1 and PHF19 increased (Kloet et al., 2016).

There are two main functional axes of primary PRC2 recruitment in mESCs, involving either recruitment through MTF2-PRC2.1 binding to DNA or JARID2-PRC2.2 binding to H2AK119ub, both of which are reinforced by H3K27me3-EED-positive feedback (Figure 5). The relative weight of these two mechanisms, however, depends on the genomic location, involving stratification of Polycomb targets into two major categories. The largest group (in this study, clusters 1–4 from Figure 2A onward) contains mainly bivalent genes with narrow H3K27me3 domains, which rely more on PRC2.1-mediated recruitment (this study and Healy et al., 2019). At these locations, MTF2 is sufficient to kickstart recruitment, which is then reinforced by the EED feedback loop and PRC2.2. Therefore, only the combination of JARID2 ablation and EED inhibition reduces recruitment to the levels mediated by MTF2 alone without core PRC2 (Figure 2A, *Eed*$^{-/-}$). Hence, the simultaneous absence of MTF2, H3K27me3, and H2AK119ub is required to abolish all core PRC2 enrichment from these regions in mESCs. The smaller group (in this study, clusters 5–6), instead relies more on PRC1 and PRC2.2, and contains very lowly expressed (in mESCs) but developmentally relevant genes, such as all the Hox genes. Here, vPRC1 activity is required to induce JARID2 and PRC2.2 recruitment, providing an alternative recruitment path to MTF2-PRC2.1 binding described above. MTF2 still binds to these locations, but likely indirectly, mediated through binding of EED in PRC2.1 to the H3K27me3 that is deposited by PRC2.2. This is supported by the loss of MTF2 in *Eed*$^{-/-}$, *Jarid2*$^{-/-}$ + EED226, and *Ring1b*$^{-/-}$ + EED226, and by the sparse presence of DNA shape-permissive GCG sequences, which are likely insufficient to achieve sustained DNA-driven MTF2 recruitment.

The observations in the current study further substantiate previous work showing that the role of PRC1 and PRC2 are largely intertwined, as both complexes can be recruited independently, but simultaneously modulate their mutual recruitment (Blackledge et al., 2014; Morey et al., 2013; Tavares et al., 2012). Our analyses of EED226-treated mESCs reveals that ~40% of PRC1 recruitment depends on the presence of H3K27me3 (Figures 4E and 4F), which likely involves canonical PRC1 (cPRC1) complexes containing CBX7 that can bind to H3K27me3 (Morey et al., 2012, 2013). The remainder (~60%) of (PRC2-independent) PRC1 comprises vPRC1 complexes containing KDM2B, that, similarly to MTF2, can bind to CG-rich DNA (Blackledge et al., 2020; Farcas et al., 2012; Fursova et al., 2019; Tamburri et al., 2020; Wu et al., 2013). Together, these observations further corroborate the hypothesis that PRC1 and PRC2 can bind autonomously, but are synergistic for their reciprocal recruitment.

Collectively, the observations here provide novel insights into Polycomb recruitment in ESCs and provide a model in which PRC2 recruitment can be initiated solely through direct recruitment via DNA, after which functional interactions between PRC2.1/PRC2.2 and PRC2.2/PRC1 are required to achieve the full establishment of Polycomb binding through self and mutual reinforcement.

EXPERIMENTAL PROCEDURES

ESC Culture

WT E14 ESCs (129/Ola background) and knockout ESCs were maintained in Dulbecco's modified Eagle medium containing 15% fetal bovine serum, 10 mM sodium pyruvate (Gibco), 5 μM beta-mercaptoethanol (Sigma) and leukemia inhibitory factor (1,000 U/mL; Millipore). To inhibit EED function, ESCs were treated with 10 μM EED226 (Qi et al., 2017) for 4 days. Complete removal of H3K27me3 was checked by western blot.

ChIP-Seq and Data Analysis

Nuclei were isolated from ESCs crosslinked in 1% PFA and sonicated using a Bioruptor Pico. ChIPs were performed overnight using protein A/G magnetic beads and specific antibodies. Eluted DNA was decrosslinked and prepared for sequencing using the Kapa HyperPrep Kit (Kapa Biosystems) using NEXTflex adapters (Bio Scientific). All ChIPs were sequenced on an Illumina NextSeq machine. Reads were aligned to the mouse genome (GRCm38/mm10). For spike-in ChIPs, reads were normalized on the *Drosophila* genome (dm6). Details can be found in the Supplemental Experimental Procedures.

Proteomics

Cell pellets were dissolved in RIPA buffer at a density of 10^4 cells per μL and briefly sonicated to ensure proper cell lysis (van Mierlo et al., 2019b). Total cell protein extracts (10 μg) or decrosslinked chromatin extracts (30 μg) were processed using Filter Aided Sample Preparation and digested overnight with trypsin. Peptide mixtures were desalted before liquid chromatography-mass spectrometry analysis. Thermo RAW files were analyzed using MaxQuant 1.5.1.0 with default settings and LFQ, IBAQ, and match between runs enabled. In Perseus, contaminant and reverse hits were filtered out. WT, MTF2 knockout, and JARID2 knockout ESCs were grouped. Only proteins that had an LFQ value in at least one of the conditions were maintained. Missing values were imputed using default settings in Perseus.

Data and Code Availability

ChIP-seq data are available via NCBI GEO (https://www.ncbi.nlm.nih.gov/geo/), accession GSE133085. A track data hub for the UCSC genome browser with the ChIP-seq data are located at the authors' website (http://veenstra.science.ru.nl/trackhubm.htm). Proteomics data can be accessed via PRIDE (https://www.ebi.ac.uk/pride/), accession PXD014290.

SUPPLEMENTAL INFORMATION

Supplemental Information can be found online at https://doi.org/10.1016/j.stemcr.2020.07.007.

AUTHOR CONTRIBUTIONS

M.P., G.v.M., H.M., and G.J.C.V. conceived the study. M.P. and G.v.M. performed the experiments, with help from C.L., S.M.T.W., and D.W.Z. M.P. performed ChIP-seq analysis. G.v.M. performed proteomic analysis. M.P., G.v.M., H.M., and G.J.C.V. wrote the manuscript. H.M. and G.J.C.V. supervised the study.

ACKNOWLEDGMENTS

This work has been financially supported by the People Program (Marie Curie Actions) of the European Union's Seventh Framework Program FP7 under grant agreement number 607142 (DevCom). G.v.M. is supported by the Oncode Institute, which is partly funded by the Dutch Cancer Society (KWF). H.M. is supported by the Netherlands Organisation for Scientific Research (NWO-VIDI 864.12.007). This work was carried out on the Dutch national e-infrastructure with the support of SURF Cooperative.

Received: June 5, 2020
Revised: July 7, 2020
Accepted: July 8, 2020
Published: August 6, 2020

REFERENCES

Beringer, M., Pisano, P., Di Carlo, V., Blanco, E., Chammas, P., Vizán, P., Gutiérrez, A., Aranda, S., Payer, B., Wierer, M., et al. (2016). EPOP functionally links Elongin and polycomb in pluripotent stem cells. Mol. Cell *64*, 645–658.

Bernstein, B.E., Mikkelsen, T.S., Xie, X., Kamal, M., Huebert, D.J., Cuff, J., Fry, B., Meissner, A., Wernig, M., Plath, K., et al. (2006). A bivalent chromatin structure marks key developmental genes in embryonic stem cells. Cell *125*, 315–326.

Blackledge, N.P., Farcas, A.M., Kondo, T., King, H.W., McGouran, J.F., Hanssen, L.L.P., Ito, S., Cooper, S., Kondo, K., Koseki, Y., et al. (2014). Variant PRC1 complex-dependent H2A ubiquitylation drives PRC2 recruitment and polycomb domain formation. Cell *157*, 1445–1459.

Blackledge, N.P., Fursova, N.A., Kelley, J.R., Huseyin, M.K., Feldmann, A., and Klose, R.J. (2020). PRC1 catalytic activity is central to polycomb system function. Mol. Cell *77*, 857–874.e9.

Brockdorff, N. (2013). Noncoding RNA and polycomb recruitment. RNA *19*, 429–442.

Brookes, E., De Santiago, I., Hebenstreit, D., Morris, K.J., Carroll, T., Xie, S.Q., Stock, J.K., Heidemann, M., Eick, D., Nozaki, N., et al. (2012). Polycomb associates genome-wide with a specific RNA polymerase II variant, and regulates metabolic genes in ESCs. Cell Stem Cell *10*, 157–170.

Casanova, M., Preissner, T., Cerase, A., Poot, R., Yamada, D., Li, X., Appanah, R., Bezstarosti, K., Demmers, J., Koseki, H., et al. (2011). Polycomblike 2 facilitates the recruitment of PRC2 Polycomb group complexes to the inactive X chromosome and to target loci in embryonic stem cells. Development *138*, 1471–1482.

Chen, S., Jiao, L., Liu, X., Yang, X., and Liu, X. (2020). A dimeric structural scaffold for PRC2-PCL targeting to CpG island chromatin. Mol. Cell *77*, 1265–1278.e7.

Conway, E., Jerman, E., Healy, E., Ito, S., Holoch, D., Oliviero, G., Deevy, O., Glancy, E., Fitzpatrick, D.J., Mucha, M., et al. (2018). A family of vertebrate-specific polycombs encoded by the LCOR/LCORL genes balance PRC2 subtype activities. Mol. Cell *70*, 408–421.e8.

Cooper, S., Dienstbier, M., Hassan, R., Schermelleh, L., Sharif, J., Blackledge, N.P., DeMarco, V., Elderkin, S., Koseki, H., Klose, R., et al. (2014). Targeting polycomb to pericentric heterochromatin in embryonic stem cells reveals a role for H2AK119u1 in PRC2 recruitment. Cell Rep. *7*, 1456–1470.

Cooper, S., Grijzenhout, A., Underwood, E., Ancelin, K., Zhang, T., Nesterova, T.B., Anil-Kirmizitas, B., Bassett, A., Kooistra, S.M., Agger, K., et al. (2016). Jarid2 binds mono-ubiquitylated H2A lysine 119 to mediate crosstalk between Polycomb complexes PRC1 and PRC2. Nat. Commun. *7*, 13661.

Farcas, A.M., Blackledge, N.P., Sudbery, I., Long, H.K., McGouran, J.F., Rose, N.R., Lee, S., Sims, D., Cerase, A., Sheahan, T.W., et al. (2012). KDM2B links the polycomb repressive complex 1 (PRC1) to recognition of CpG islands. eLife *1*. https://doi.org/10.7554/eLife.00205.

Faust, C., Lawson, K.A., Schork, N.J., Thiel, B., and Magnuson, T. (1998). The Polycomb-group gene EED is required for normal morphogenetic movements during gastrulation in the mouse embryo. Development *125*, 4495–4506.

Ferrari, K.J., Scelfo, A., Jammula, S.G., Cuomo, A., Barozzi, I., Stützer, A., Fischle, W., Bonaldi, T., and Pasini, D. (2014). Polycomb-dependent H3K27me1 and H3K27me2 regulate active transcription and enhancer fidelity. Mol. Cell *53*, 49–62.

Fursova, N.A., Blackledge, N.P., Nakayama, M., Ito, S., Koseki, Y., Farcas, A.M., King, H.W., Koseki, H., and Klose, R.J. (2019). Synergy between variant PRC1 complexes defines polycomb-mediated gene repression. Mol. Cell *74*, 1020–1036.e8.

Grijzenhout, A., Godwin, J., Koseki, H., Gdula, M.R., Szumska, D., McGouran, J.F., Bhattacharya, S., Kessler, B.M., Brockdorff, N., and Cooper, S. (2016). Functional analysis of AEBP2, a PRC2 Polycomb protein, reveals a Trithorax phenotype in embryonic development and in ESCs. Development *143*, 2716–2723.

Hauri, S., Comoglio, F., Seimiya, M., Gerstung, M., Glatter, T., Hansen, K., Aebersold, R., Paro, R., Gstaiger, M., and Beisel, C. (2016). A high-density map for navigating the human polycomb complexome. Cell Rep. *17*, 583–595.

Healy, E., Mucha, M., Glancy, E., Fitzpatrick, D.J., Conway, E., Neikes, H.K., Monger, C., Van Mierlo, G., Baltissen, M.P., Koseki, Y., et al. (2019). PRC2.1 and PRC2.2 synergize to coordinate H3K27 trimethylation. Mol. Cell *76*, 437–452.e6.

Herz, H.-M., Mohan, M., Garrett, A.S., Miller, C., Casto, D., Zhang, Y., Seidel, C., Haug, J.S., Florens, L., Washburn, M.P., et al. (2012). Polycomb repressive complex 2-dependent and -independent functions of Jarid2 in transcriptional regulation in *Drosophila*. Mol. Cell. Biol. *32*, 1683–1693.

Højfeldt, J.W., Laugesen, A., Willumsen, B.M., Damhofer, H., Hedehus, L., Tvardovskiy, A., Mohammad, F., Jensen, O.N., and Helin, K. (2018). Accurate H3K27 methylation can be established de novo by SUZ12-directed PRC2. Nat. Struct. Mol. Biol. *25*, 225–232.

Højfeldt, J.W., Hedehus, L., Laugesen, A., Tatar, T., Wiehle, L., and Helin, K. (2019). Non-core subunits of the PRC2 complex are collectively required for its target-site specificity. Mol. Cell *76*, 423–436.e3.

Isono, K., Endo, T.A., Ku, M., Yamada, D., Suzuki, R., Sharif, J., Ishikura, T., Toyoda, T., Bernstein, B.E., and Koseki, H. (2013). SAM domain polymerization links subnuclear clustering of PRC1 to gene silencing. Dev. Cell *26*, 565–577.

Kalb, R., Latwiel, S., Baymaz, H.I., Jansen, P.W.T.C., Müller, C.W., Vermeulen, M., and Müller, J. (2014). Histone H2A monoubiquitination promotes histone H3 methylation in Polycomb repression. Nat. Struct. Mol. Biol. *21*, 569–571.

Kaneko, S., Son, J., Bonasio, R., Shen, S.S., and Reinberg, D. (2014). Nascent RNA interaction keeps PRC2 activity poised and in check. Genes Dev. *28*, 1983–1988.

Kloet, S.L., Makowski, M.M., Baymaz, H.I., van Voorthuijsen, L., Karemaker, I.D., Santanach, A., Jansen, P.W.T.C., Di Croce, L., and Vermeulen, M. (2016). The dynamic interactome and genomic targets of Polycomb complexes during stem-cell differentiation. Nat. Struct. Mol. Biol. *23*, 682–690.

Landeira, D., Sauer, S., Poot, R., Dvorkina, M., Mazzarella, L., Jørgensen, H.F., Pereira, C.F., Leleu, M., Piccolo, F.M., Spivakov, M., et al. (2010). Jarid2 is a PRC2 component in embryonic stem cells required for multi-lineage differentiation and recruitment of PRC1 and RNA polymerase II to developmental regulators. Nat. Cell Biol. *12*, 618–624.

Laprell, F., Finkl, K., and Müller, J. (2017). Propagation of Polycomb-repressed chromatin requires sequence-specific recruitment to DNA. Science *356*, 85–88.

Lau, M.S., Schwartz, M.G., Kundu, S., Savol, A.J., Wang, P.I., Marr, S.K., Grau, D.J., Schorderet, P., Sadreyev, R.I., Tabin, C.J., et al. (2017). Mutation of a nucleosome compaction region disrupts Polycomb-mediated axial patterning. Science *355*, 1081–1084.

Li, G., Margueron, R., Ku, M., Chambon, P., Bernstein, B.E., and Reinberg, D. (2010). Jarid2 and PRC2, partners in regulating gene expression. Genes Dev. *24*, 368–380.

Li, H., Liefke, R., Jiang, J., Kurland, J.V., Tian, W., Deng, P., Zhang, W., He, Q., Patel, D.J., Bulyk, M.L., et al. (2017). Polycomb-like proteins link the PRC2 complex to CpG islands. Nature *549*, 287–291.

Liefke, R., Karwacki-Neisius, V., and Shi, Y. (2016). EPOP interacts with elongin BC and USP7 to modulate the chromatin landscape. Mol. Cell *64*, 659–672.

Long, H.K., Sims, D., Heger, A., Blackledge, N.P., Kutter, C., Wright, M.L., Grützner, F., Odom, D.T., Patient, R., Ponting, C.P., et al. (2013). Epigenetic conservation at gene regulatory elements revealed by non-methylated DNA profiling in seven vertebrates. eLife *2*, e00348.

Margueron, R., Justin, N., Ohno, K., Sharpe, M.L., Son, J., Drury, W.J., Voigt, P., Martin, S.R., Taylor, W.R., De Marco, V., et al. (2009). Role of the polycomb protein EED in the propagation of repressive histone marks. Nature *461*, 762–767.

van Mierlo, G., Veenstra, G.J.C., Vermeulen, M., and Marks, H. (2019a). The complexity of PRC2 subcomplexes. Trends Cell Biol. *29*, 660–671.

van Mierlo, G., Dirks, R.A.M., De Clerck, L., Brinkman, A.B., Huth, M., Kloet, S.L., Saksouk, N., Kroeze, L.I., Willems, S., Farlik, M., et al. (2019b). Integrative proteomic profiling reveals PRC2-dependent epigenetic crosstalk maintains ground-state pluripotency. Cell Stem Cell *24*, 123–137.e8.

Morey, L., Pascual, G., Cozzuto, L., Roma, G., Wutz, A., Benitah, S.A., and Di Croce, L. (2012). Nonoverlapping functions of the polycomb group Cbx family of proteins in embryonic stem cells. Cell Stem Cell *10*, 47–62.

Morey, L., Aloia, L., Cozzuto, L., Benitah, S.A., and Di Croce, L. (2013). RYBP and Cbx7 define specific biological functions of polycomb complexes in mouse embryonic stem cells. Cell Rep. *3*, 60–69.

O'Carroll, D., Erhardt, S., Pagani, M., Barton, S.C., Surani, M.A., and Jenuwein, T. (2001). The polycomb-group gene Ezh2 is required for early mouse development. Mol. Cell. Biol. *21*, 4330–4336.

Oksuz, O., Narendra, V., Lee, C.-H., Descostes, N., LeRoy, G., Raviram, R., Blumenberg, L., Karch, K., Rocha, P.P., Garcia, B.A., et al. (2018). Capturing the onset of PRC2-mediated repressive domain formation. Mol. Cell *70*, 1149–1162.e5.

Pasini, D., Bracken, A.P., Hansen, J.B., Capillo, M., and Helin, K. (2007). The polycomb group protein Suz12 is required for embryonic stem cell differentiation. Mol. Cell. Biol. *27*, 3769–3779.

Pasini, D., Cloos, P.A.C., Walfridsson, J., Olsson, L., Bukowski, J.P., Johansen, J.V., Bak, M., Tommerup, N., Rappsilber, J., and Helin, K. (2010). JARID2 regulates binding of the Polycomb repressive complex 2 to target genes in ES cells. Nature *464*, 306–310.

Pengelly, A.R., Copur, Ö., Jäckle, H., Herzig, A., and Müller, J. (2013). A histone mutant reproduces the phenotype caused by loss of histone-modifying factor Polycomb. Science *339*, 698–699.

Perino, M., van Mierlo, G., Karemaker, I.D., van Genesen, S., Vermeulen, M., Marks, H., van Heeringen, S.J., and Veenstra, G.J.C. (2018). MTF2 recruits Polycomb Repressive Complex 2 by helical-shape-selective DNA binding. Nat. Genet. *50*, 1002–1010.

Poepsel, S., Kasinath, V., and Nogales, E. (2018). Cryo-EM structures of PRC2 simultaneously engaged with two functionally distinct nucleosomes. Nat. Struct. Mol. Biol. *25*, 154–162.

Qi, W., Zhao, K., Gu, J., Huang, Y., Wang, Y., Zhang, H., Zhang, M., Zhang, J., Yu, Z., Li, L., et al. (2017). An allosteric PRC2 inhibitor targeting the H3K27me3 binding pocket of EED. Nat. Chem. Biol. *13*, 381–388.

Sanulli, S., Justin, N., Teissandier, A., Ancelin, K., Portoso, M., Caron, M., Michaud, A., Lombard, B., da Rocha, S.T., Offer, J., et al. (2015). Jarid2 methylation via the PRC2 complex regulates H3K27me3 deposition during cell differentiation. Mol. Cell *57*, 769–783.

Smits, A.H., Jansen, P.W.T.C., Poser, I., Hyman, A.A., and Vermeulen, M. (2013). Stoichiometry of chromatin-associated protein complexes revealed by label-free quantitative mass spectrometry-based proteomics. Nucleic Acids Res. *41*, e28.

Son, J., Shen, S.S., Margueron, R., and Reinberg, D. (2013). Nucleosome-binding activities within JARID2 and EZH1 regulate the function of PRC2 on chromatin. Genes Dev. *27*, 2663–2677.

Tamburri, S., Lavarone, E., Fernández-Pérez, D., Conway, E., Zanotti, M., Manganaro, D., and Pasini, D. (2020). Histone H2AK119 mono-ubiquitination is essential for polycomb-mediated transcriptional repression. Mol. Cell *77*, 840–856.e5.

Tavares, L., Dimitrova, E., Oxley, D., Webster, J., Poot, R., Demmers, J., Bezstarosti, K., Taylor, S., Ura, H., Koide, H., et al. (2012). RYBP-PRC1 complexes mediate H2A ubiquitylation at polycomb target sites independently of PRC2 and H3K27me3. Cell *148*, 664–678.

Wu, X., Johansen, J.V., and Helin, K. (2013). Fbxl10/Kdm2b recruits polycomb repressive complex 1 to CpG islands and regulates H2A ubiquitylation. Mol. Cell *49*, 1134–1146.

Stem Cell Reports, Volume *15*

Supplemental Information

Two Functional Axes of Feedback-Enforced PRC2 Recruitment in Mouse Embryonic Stem Cells

Matteo Perino, Guido van Mierlo, Chet Loh, Sandra M.T. Wardle, Dick W. Zijlmans, Hendrik Marks, and Gert Jan C. Veenstra

Supplemental Information

Two functional axes of positive feedback-enforced PRC2 recruitment in mouse embryonic stem cells

Matteo Perino*, Guido van Mierlo*, Chet Loh, Sandra M.T. Wardle, Dick Zijlmans, Hendrik Marks# & Gert Jan C. Veenstra#

Contents

• Supplemental Figures S1-S5
• Supplemental Tables: Legends corresponding to Table S1 and S2
• Supplemental Experimental Procedures
• Supplemental References

Figure S1

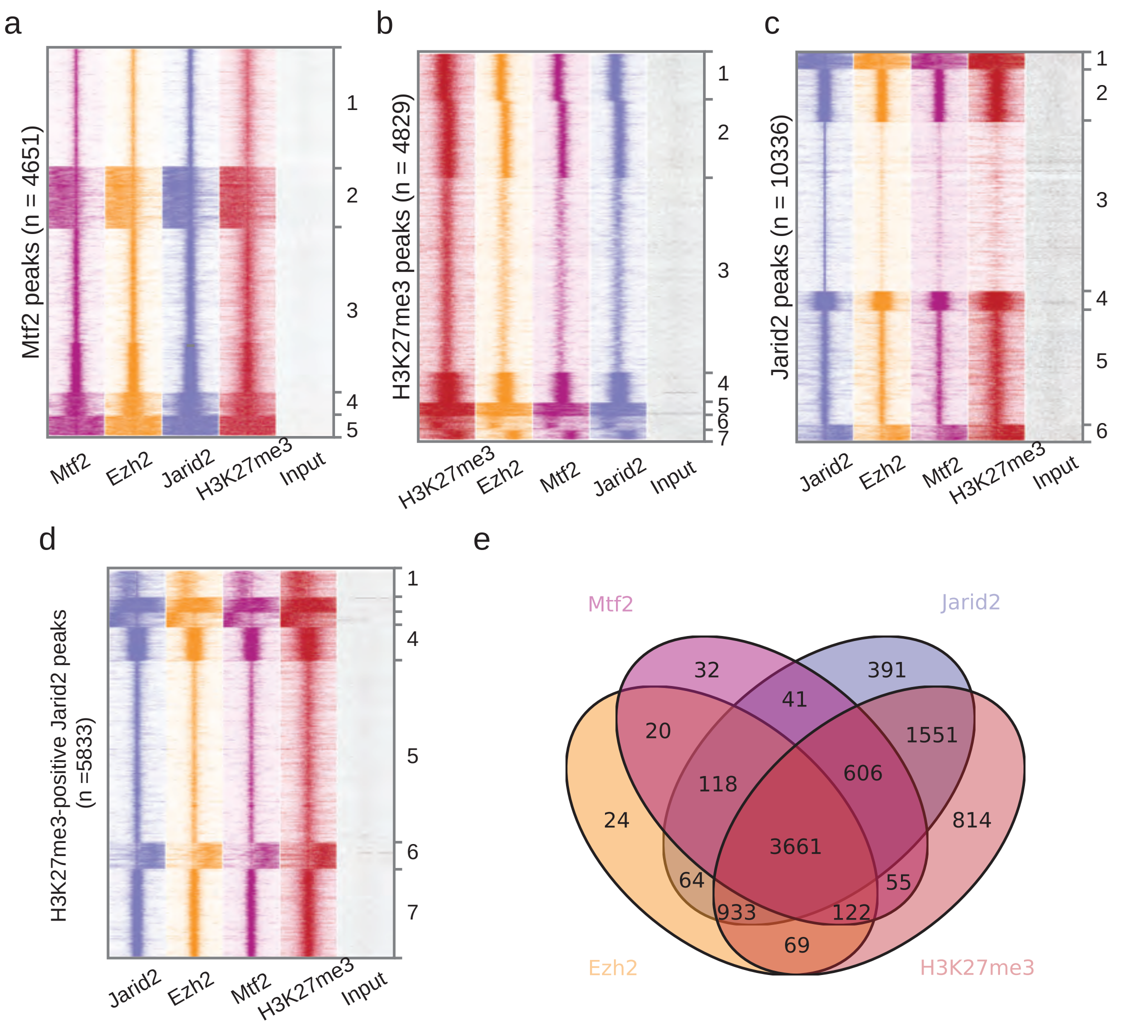

Supplementary Figure 1. a-d) Heatmap of WT ChIP-seq signal on the indicated peak set. H3K27me3-negative JARID2 peaks were excluded from further analysis. **e)** Venn diagram showing the overlap of peaks called for the ChIP-Seq of each protein independently. The ChIP-seq data represent two combined replicates from independent experiments.

Figure S2

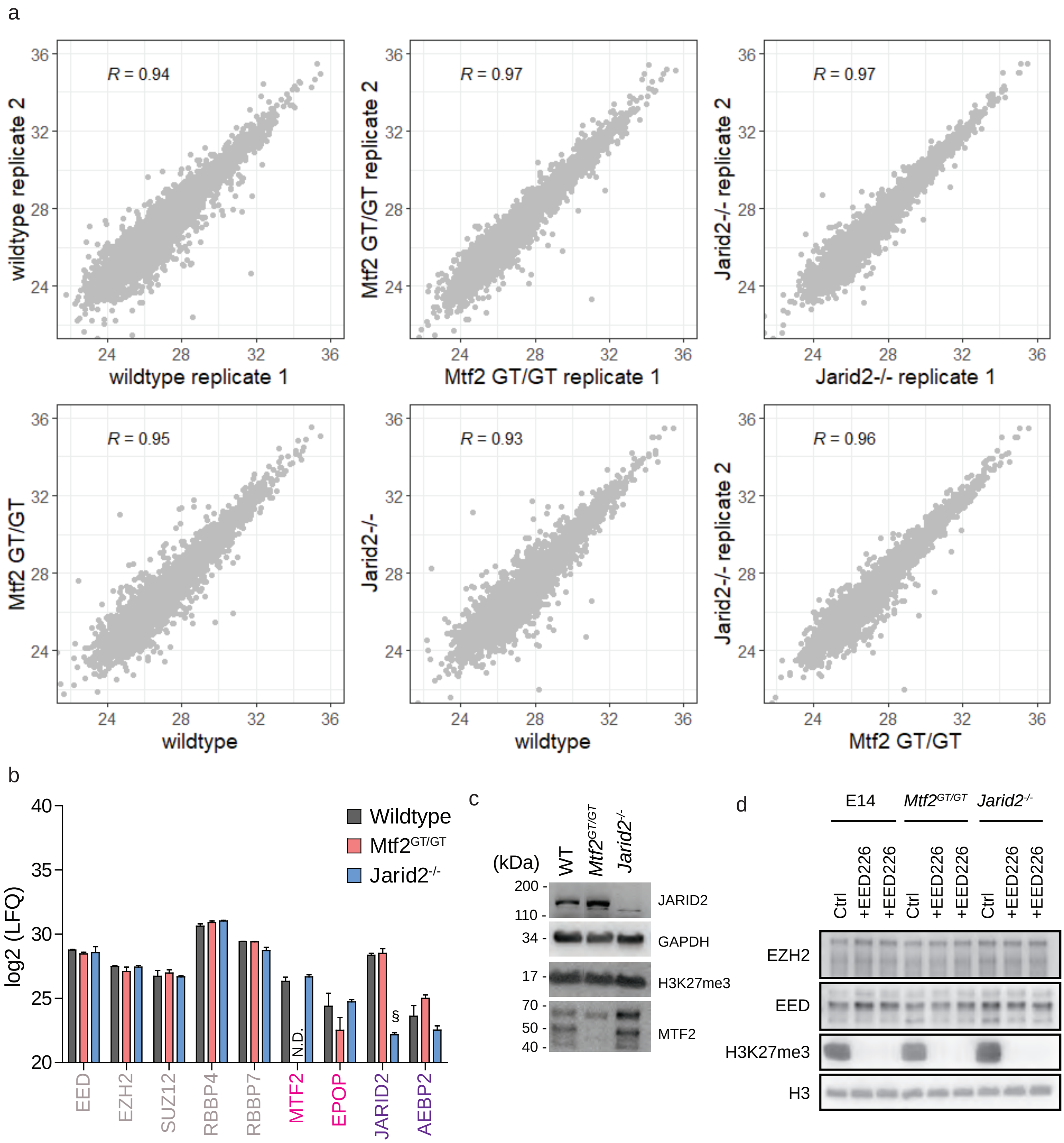

Supplementary Figure 2. a) Scatterplot of total proteome quantification across WT, Mtf2$^{GT/GT}$ and Jarid2$^{-/-}$ mESC. **b)** Quantification of the data shown in (a). § indicate detection of three residual JARID2 peptides in Jarid2$^{-/-}$. The data represent two combined replicates from independent experiments. **c)** Western blot validation of Mtf2 and Jarid2 mutant showing no residual protein. **d)** Western blot validation of H3K27me3 depletion in EED226 treated cells.

Figure S3

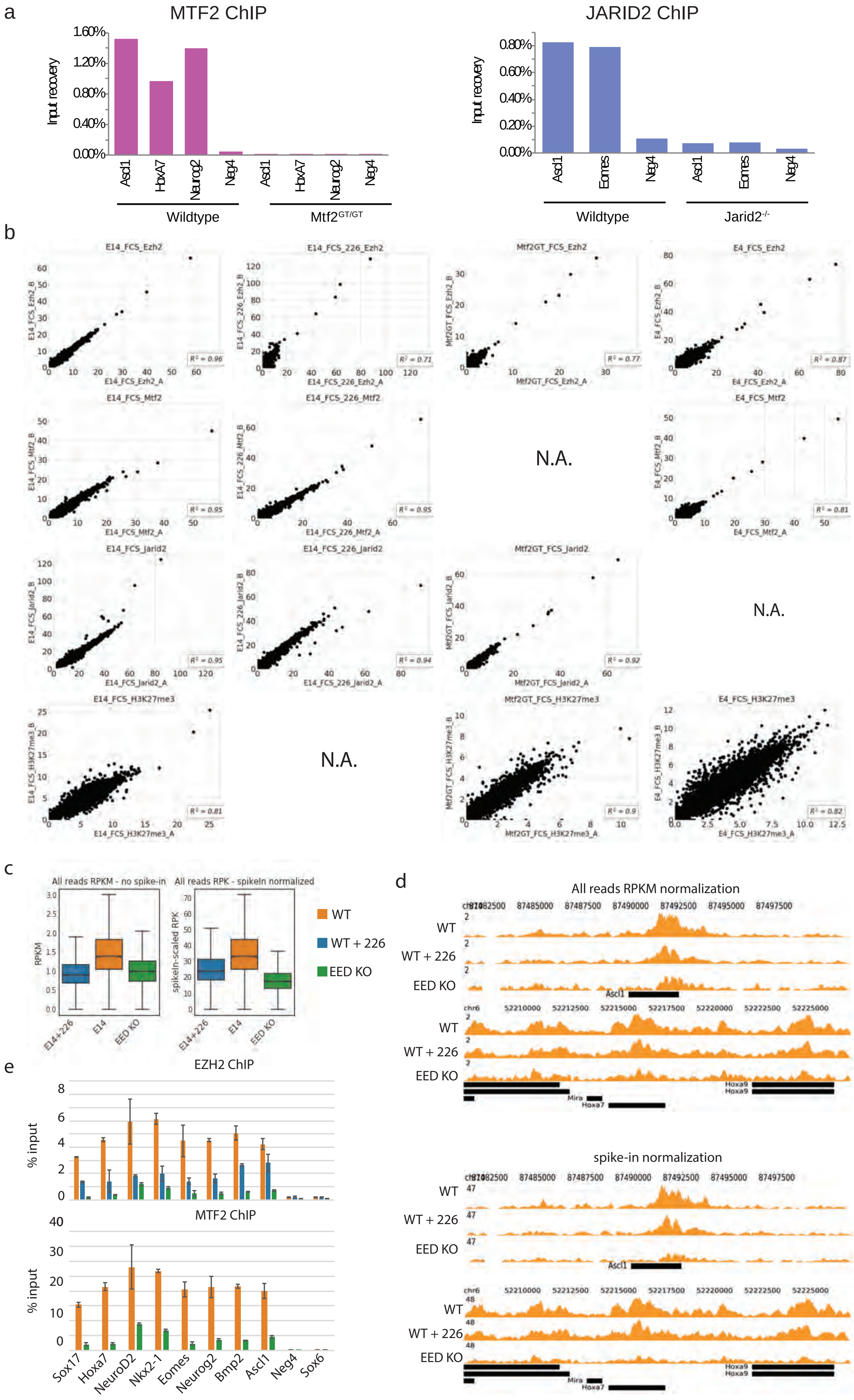
a
MTF2 ChIP
Input recovery
1.60%
1.20%
0.80%
0.40%
0.00%
Ascl1
HoxA7
Neurog2
Neg4
Wildtype
Mtf2GT/GT
JARID2 ChIP
0.80%
0.60%
0.40%
0.20%
0.00%
Eomes
Jarid2-/-
b
E14_FCS_Ezh2
E14_FCS_226_Ezh2
Mtf2GT_FCS_Ezh2
E4_FCS_Ezh2
E14_FCS_Mtf2
E14_FCS_226_Mtf2
N.A.
E4_FCS_Mtf2
E14_FCS_Jarid2
E14_FCS_226_Jarid2
Mtf2GT_FCS_Jarid2
N.A.
E14_FCS_H3K27me3
N.A.
Mtf2GT_FCS_H3K27me3
E4_FCS_H3K27me3
R² = 0.96
R² = 0.71
R² = 0.77
R² = 0.87
R² = 0.95
R² = 0.95
R² = 0.81
R² = 0.95
R² = 0.94
R² = 0.92
R² = 0.81
R² = 0.9
R² = 0.82
c
All reads RPKM - no spike-in
All reads RPK - spikeIn normalized
RPKM
spikeIn-scaled RPK
E14+226
E14
EED KO
WT
WT + 226
EED KO
d
All reads RPKM normalization
WT
WT + 226
EED KO
Ascl1
chr6
Hoxa9
Mira
Hoxa7
spike-in normalization
e
EZH2 ChIP
% input
MTF2 ChIP
Sox17
Hoxa7
NeuroD2
Nkx2-1
Eomes
Neurog2
Bmp2
Ascl1
Neg4
Sox6

Supplementary Figure 3. a) qPCR validation of MTF2 and JARID2 mutants in the respective mutants. **b)** Scatterplot of peak RPKM showing high reproducibility of ChIP replicates. **c)** Peak intensity distribution of spike-in EZH2 ChIP-seq normalized with either RPKM or spike-in correction. Boxplots represent median and interquartile range (IQR; whiskers, 1.5 IQR). Outliers not shown. **d)** Examples of EZH2 ChIP-seq of the loci quantified in c). **e)** qPCR quantification of ChIP for EZH2 and MTF2 on multiple loci (n=2, independent experiments). Error bars represent standard deviation.

Figure S4

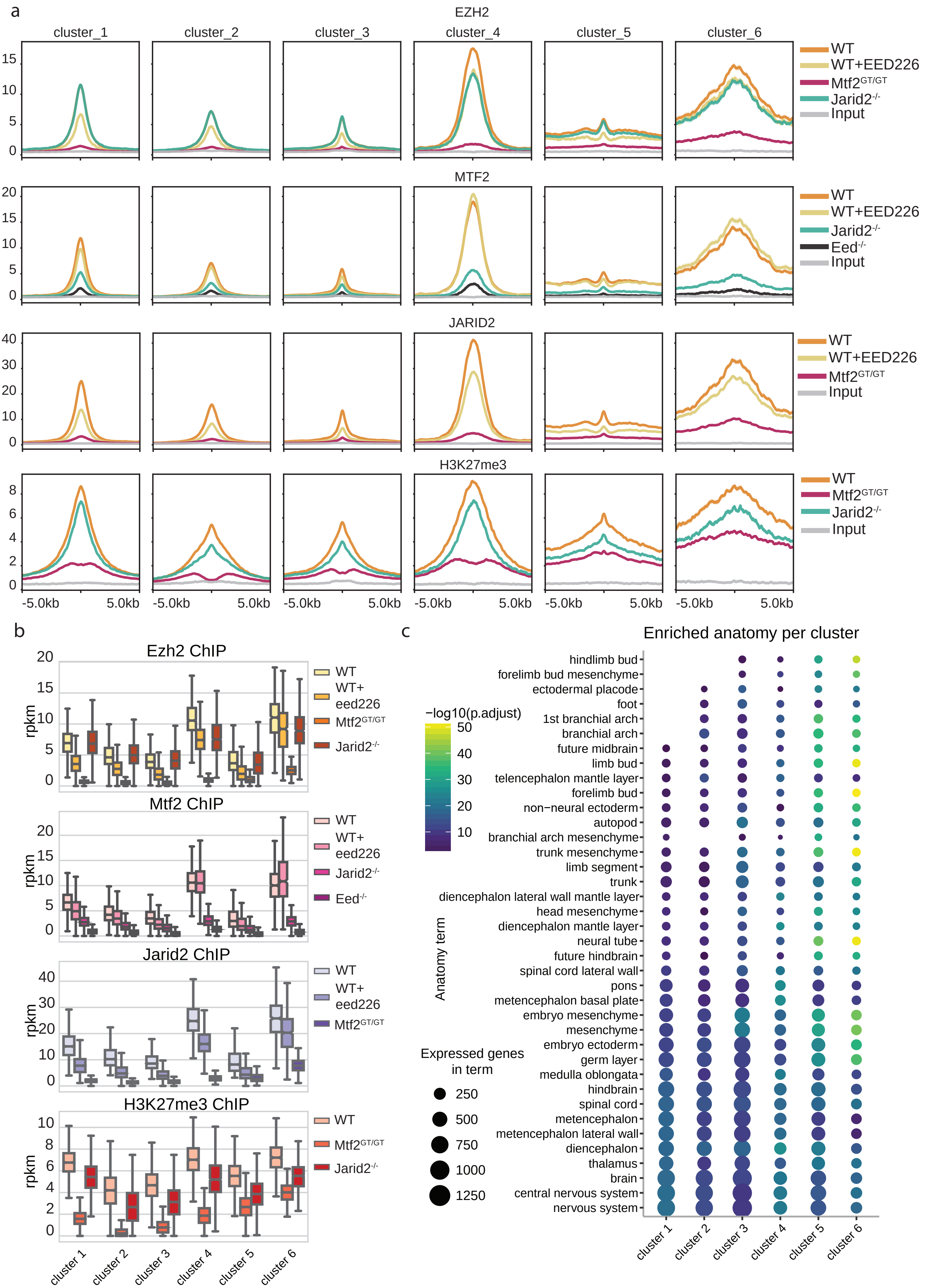
a
EZH2
cluster_1
cluster_2
cluster_3
cluster_4
cluster_5
cluster_6
WT
WT+EED226
Mtf2GT/GT
Jarid2-/-
Input
MTF2
Eed-/-
JARID2
H3K27me3
-5.0kb
5.0kb
b
Ezh2 ChIP
Mtf2 ChIP
Jarid2 ChIP
H3K27me3 ChIP
rpkm
WT+ eed226
cluster 1
cluster 2
cluster 3
cluster 4
cluster 5
cluster 6
c
Enriched anatomy per cluster
−log10(p.adjust)
Expressed genes in term
Anatomy term
hindlimb bud
forelimb bud mesenchyme
ectodermal placode
foot
1st branchial arch
branchial arch
future midbrain
limb bud
telencephalon mantle layer
forelimb bud
non−neural ectoderm
autopod
branchial arch mesenchyme
trunk mesenchyme
limb segment
trunk
diencephalon lateral wall mantle layer
head mesenchyme
diencephalon mantle layer
neural tube
future hindbrain
spinal cord lateral wall
pons
metencephalon basal plate
embryo mesenchyme
mesenchyme
embryo ectoderm
germ layer
medulla oblongata
hindbrain
spinal cord
metencephalon
metencephalon lateral wall
diencephalon
thalamus
brain
central nervous system
nervous system

Supplementary Figure 4. **a)** Average plot of the ChIP signal shown in Fig 2a, centred on called peaks. **b)** Boxplot quantification of the signal shown in Fig. 2a. Boxplots represent the median and interquartile range (IQR; whiskers, 1.5 IQR). Outliers not shown. **c)** Enrichment of anatomical terms in the genes associated with peaks in the six clusters shown in Fig 2a. Enrichment over all genes. The ChIP-seq data represent two combined replicates from independent experiments.

Figure S5

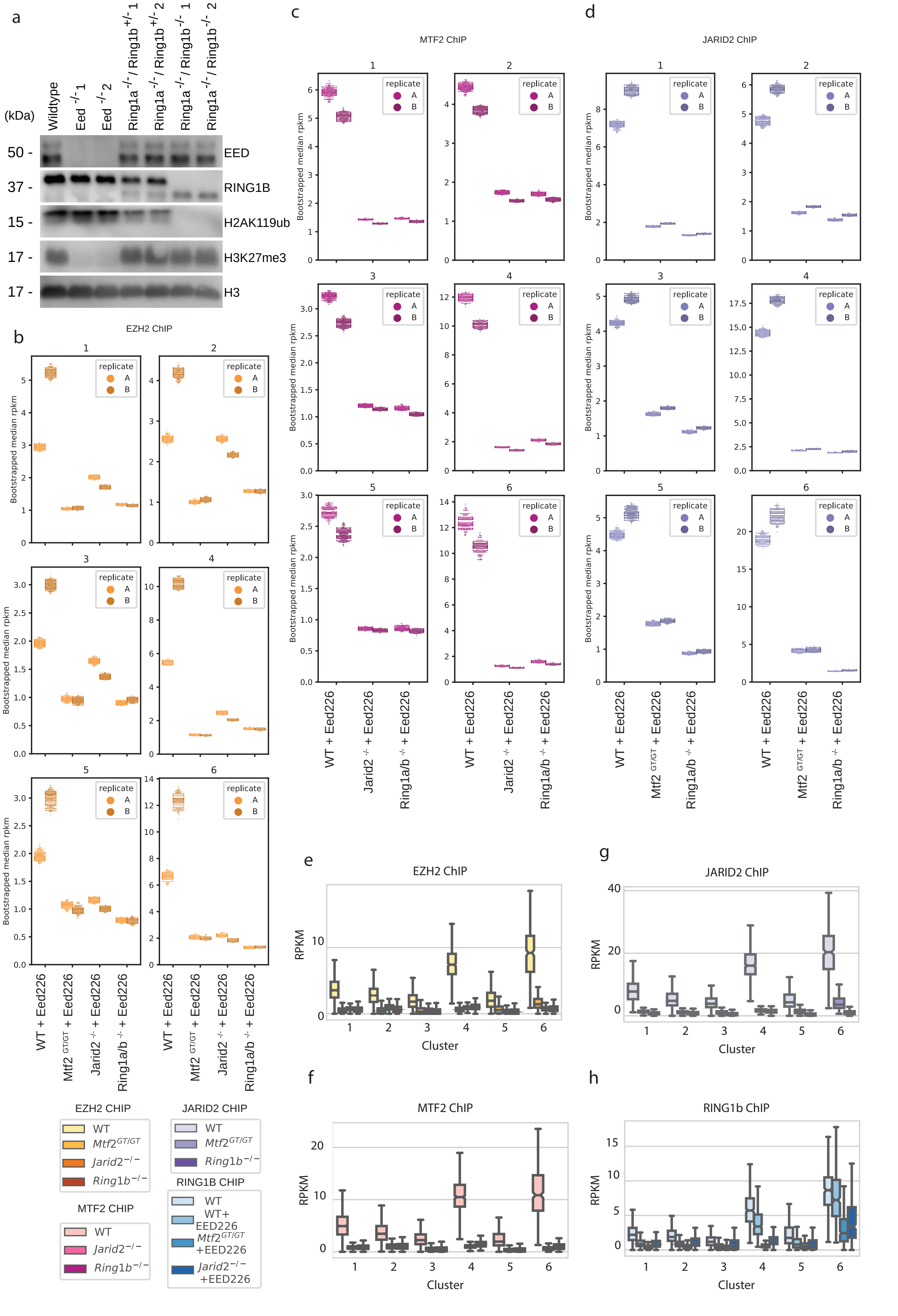
a
(kDa)
Wildtype
Eed -/- 1
Eed -/- 2
Ring1a -/- / Ring1b +/- 1
Ring1a -/- / Ring1b +/- 2
Ring1a -/- / Ring1b -/- 1
Ring1a -/- / Ring1b -/- 2
50 -
EED
37 -
RING1B
15 -
H2AK119ub
17 -
H3K27me3
17 -
H3
b
EZH2 ChIP
c
MTF2 ChIP
d
JARID2 ChIP
replicate
A
B
Bootstrapped median rpkm
WT + Eed226
Mtf2 GT/GT + Eed226
Jarid2 -/- + Eed226
Ring1a/b -/- + Eed226
e
EZH2 ChIP
f
MTF2 ChIP
g
JARID2 ChIP
h
RING1b ChIP
RPKM
Cluster
EZH2 CHIP
WT
Mtf2 GT/GT
Jarid2 -/-
Ring1b -/-
JARID2 CHIP
WT
Mtf2 GT/GT
Ring1b -/-
MTF2 CHIP
WT
Jarid2 -/-
Ring1b -/-
RING1B CHIP
WT
WT+ EED226
Mtf2 GT/GT +EED226
Jarid2 -/- +EED226

Supplementary Figure 5. a) Western blot validation of Eed$^{-/-}$ and Ring1ab$^{-/-}$ lines. **b-d)** Bootstrapping-based RPKM quantification (methods) of the signal in Fig 4 a-d. Each colored dot represents the median of one round of bootstrapping. Replicates are plotted independently. **e-h)** Boxplot quantification of data in Fig 4. Boxplots represent median and interquartile range (IQR; 763 whiskers, 1.5 IQR). Outliers not shown. Annotation of the colours is presented on the left of the boxplots. The data represent two combined replicates from independent experiments.

Supplemental Tables

Supplemental Table S1 and Table S2 are provided as Excel files and contain the following information:

- **Table S1:** Total proteome quantification
- **Table S2:** Chromatin proteome quantification (ChEP)

Supplemental experimental procedures

Embryonic stem cell culture

Eed-/- ESCs have been described by Schoeftner et al., 2006, Jarid2-/- ESCs have been described in Landeira et al. (Landeira et al., 2010). Mtf2 knockout (Mtf2GT/GT) (Li et al., 2011) and Ring1a-/- / Ring1b+/- ESCs (Endoh et al., 2008) were a kind gift from Haruhiko Koseki. Ring1b ESCs are knockout for Ring1a and trans-heterozygous for Ring1b (null/floxed). Full knockout of Ring1b was induced through treatment with Tamoxifen (OHT) for 2 days. All knockout ESCs were validated using western blot.

Western blot and antibodies

Cell pellets were dissolved in RIPA buffer at a density of 104 cells per µl and briefly sonicated to ensure proper cell lysis. Proteins denatured in SDS-PAGE gels were transferred onto PVDF membranes. Primary antibodies used were rabbit anti-MTF2 (ProteinTech; 16208-1-AP), rabbit anti-JARID2 (Novus Bio; NB100-2214), rabbit anti-H3K27me3 (Millipore; 07-449), rabbit anti-H3 (Abcam;1791). Secondary antibodies were HRP-conjugated anti-rabbit (Dako; P0217) and anti-mouse (Dako; P0161). Protein bands were visualized using Pierce ECL western blotting substrate (Thermo). Images were analyzed using ImageJ.

ChIP-sequencing

Cells were crosslinked in 1% PFA at room temperature for 8-10 min. The crosslinking reaction was quenched using 1.25M glycine and cells were harvested by scraping in buffer B (0.25% Triton X-100, 10 mM EDTA, 0.5 mM EGTA, 20 mM HEPES). The suspension was centrifuged for 5 min at 1600 rpm, 4 ˚C and the pellet was resuspended in 30 ml buffer C (150 mM, 1 mM EDTA, 0.5 mM EGTA, 50 mM HEPES) and rotated for 10 min at 4 ˚C. The nuclei were centrifuged 5 min at 1600 rpm, 4 ˚C and resuspended in incubation buffer (0.15% SDS, 1% Triton X-100, 150 mM NaCl, 1 mM EDTA, 0.5 mM EGTA, 20 mM HEPES) supplemented with Protease inhibitor. Nuclei were sonicated using a Biorupter Pico to obtain chromatin with an enriched DNA length of 300 bp. The chromatin was snap-frozen and stored at -80 ˚C until further use. For ChIP, sonicated chromatin was incubated overnight with the required antibody and pulled down using protein A/G magnetic beads (Perino et al., 2018). All ChIPs were performed using 3 µl/sample of the following antibodies: MTF2 (Aviva System Biology ARP34292, lot QC49692-42166), H3K27me3 (Millipore 07-449, lot 2717675), EZH2 (Diagenode C15410039, lot 003), JARID2 (Novus Biologicals NB100-2214, Lot E2), RING1B (Abcam, AB3832 lot GR86503-25) and spike in antibody (Active Motif 61686, lot 00419007).
For Spike-in ChIPs, 50ng of spike-in chromatin (*Drosophila melanogaster*) and a *Drosophila melanogaster*-specific H2Av antibody (Active Motif #61686, 2ug per chip) were added to selected chips. After washes, eluted chromatin was de-crosslinked overnight and purified with MinElute PCR Purification columns (Qiagen). After qPCR quality check for target enrichment, up to 5 ng/sample of ChIP was prepared for sequencing using the Kapa Hyper-prep Kit (Kapa Biosystems) using NEXTflex adapters (Bio Scientific), followed by 8-12 cycles amplification by PCR. After size-selection using E-gel (Invitrogen) or KAPA beads (Kapa Biosystems) to enrich for 300bp fragments, libraries were sequenced paired-end on an Illumina NextSeq500. qPCR analysis of ChIP DNA was performed with iQ SYBR Green Supermix (Bio-Rad) on a CFX96 Real-Time System C1000 Thermal Cycler (Bio-Rad). All the ChIP-Seq experiments in this study were performed at least in duplicate, from independent chromatin preparations.

ChIP-sequencing data analysis

Newly generated and previously published data sets (Perino et al., 2018) were processed in parallel with identical settings. To ensure maximum comparability (75bp single-end vs 42bp paired-end) and accurate quantification, all fastq files were trimmed to 42bp using fastx_trimmer (version 0.0.13.2), and in case of paired-end sequencing, only read_1 was used for analysis. All fastq files were mapped using bwa (version 0.7.10-r789), filtered to retain only uniquely mapping reads using mapping quality of 30 and samtools (version 1.7, flag -F 1024), then normalized for sequencing depth to produce bigwig. Peaks were called with MACS2-2.7 (Zhang et al., 2008) with qvalue 0.0001 using --call-summits for transcription factors and --broad for H3K27me3. Only peaks independently called in both replicates were used for downstream analysis. High-confidence peaks for each mark were obtained by merging peaks called in both replicates and overlapping by at least 50% of their length, and combined to obtain the list of all PRC2 peaks. Heatmaps of ChIP-Seq signals were generated using fluff v3.0.2 (Georgiou and van Heeringen, 2016) with the following settings. Bam files were used as input, the clustering method was set to k-means, the -r option was enabled for read-depth normalization (for non-spike in data). The heatmaps were clustered for dynamics using the "–g" option. Specifically, this -g option allows identifying dynamic patterns specifically at peak centers, for which a distance of 1kb in each direction from the peak center

was used in combination with Pearson correlation similarity as a distance metric. In contrast to the most common approach using Euclidian distance metrics across a wider area (typically +/- 5kb), this setting removes the influence of peak flanking regions on clustering, resulting in a clusters that reflect the peaks intensity at the summit rather than the general shape of the signal over several kb, thus better identifying dynamic changes across conditions at the most strongly bound region. As the peak flanking regions are ignored during clustering and the peak center considered as a single bin, information about the directionality of the signal outside the peak is not considered for clustering. ChIP metaplots were obtained with deeptools v 3.1.3 (Ramírez et al., 2016). Anatomy term enrichment was calculated using MouseMine (Motenko et al., 2015). RPKM bootstrapping analysis was performed using scipy (v 1.1.0). RPKM from the two independent ChIP-seq replicates were combined into a single pool. Values were drawn from this pool, recorded, and returned, such that every value could be drawn multiple times. For each bootstrapping round, a number of values matching the total number of PRC2 peaks was drawn, and the median plotted as one dot in the swarm plot. Confidence intervals (99.9%) were calculated from 100 bootstrapping events. DNA shape analysis was performed using the DNAshape package (Zhou et al., 2013).

ChIP-seq samples supplemented with *Drosophila* chromatin as spike-in were mapped on a combined mm10-dm6 genome, and reads mapping on multiple genomic locations or representing PCR duplicates were filtered out. The resulting bam files were split according to the species of origin and dm6 reads were used to calculate a per-sample scaling factor relative to the less deeply sequenced sample. ChIP-seq were first normalized based on the number of spike-in reads to obtain the number of reads per million of spike-in reads. This is achieved calculating a NormFactor with:

[1] NormFactor = 1e-6 * reads_ChIP_dm6

To account for the potentially varying starting amount of spike-in in different samples, we calculate F, the fraction of spike-in reads in input samples:

[2] F = Input_dm6 / (Input_dm6 + Input_mm10)

The final scaling factor S is calculated dividing the NormFactor of each sample by the F of its input:

[3] S = NormFactor / F

S is then scaled to obtain min(S) = 1.

[4] Scaled_S = S/min(S)

For comparison of peak intensity in RPKM and spike-in normalizations, reads per peak per kb (RPK) were calculated from the bam files and then normalized for either sequencing depth (RPKM) or spike-in scaling factor. This scaling factor was also used to down sample the bam files containing the mm10 reads used to produce the spike-in normalized heatmaps.

Chromatin Enrichment for Proteomics (ChEP)

Chromatin enrichment was performed as described previously, with minor adaptations (van Mierlo et al., 2019). In short, cells were crosslinked in 1% PFA for 10 minutes at 37 ˚C, washed twice in PBS, scraped and transferred to 2 ml tubes. Cells were resuspended in 1 ml ice-cold cell lysis buffer (25 mm TRIS pH 7.4, 0.1% Triton X-100, 85 mM KCl, 1X Roche protease inhibitor) and centrifuged at 2,300g for 5 min at 4 °C. The supernatant (cytoplasmic fraction) was removed and cell pellets were resuspended in 500 µl SDS buffer (10 mM TRIS pH 7.4, 10 mM EDTA, 4% SDS, 1X Roche protease inhibitor), incubated at RT for 10 minutes, topped up to 2 ml with Urea buffer (10 mM TRIS pH 7.4, 1 mM EDTA, 8 M urea) and centrifuged at 16,100g for 30 min at RT. The supernatant was discarded and this step repeated once. Next, the pellet was resuspended in 500 µl SDS buffer, topped up to 2 ml with SDS buffer and centrifuged at 16,100g for 30 min at RT. The cell pellet was taken up in storage buffer (10 mM TRIS pH 7.4, 1 mM EDTA, 25 mM NaCl, 10% glycerol, 1X Roche protease inhibitor) and sonicated in an NGS Bioruptor (Diagenode) to solubilize the pellet. The concentration of the resulting lysate was measured using a Qubit assay (Invitrogen). For sample preparation for mass spectrometry, 30 µg of protein extract was decrosslinked for 30 minutes at 95 ˚C by adding 4X decrosslinking buffer (250 mM Tris pH8.8, 2%SDS, 0.5M 2-mercaptoethanol) to final 1X. Decrosslinked chromatin extracts were processed and analyzed the same as whole-cell proteomes.

Supplementary references

Endoh, M., Endo, T.A., Endoh, T., Fujimura, Y., Ohara, O., Toyoda, T., Otte, A.P., Okano, M., Brockdorff, N., Vidal, M., et al. (2008). Polycomb group proteins Ring1A/B are functionally linked to the core transcriptional

regulatory circuitry to maintain ES cell identity. Development *135*, 1513–1524.

Georgiou, G., and van Heeringen, S.J. (2016). fluff: exploratory analysis and visualization of high-throughput sequencing data. PeerJ *4*, e2209.

Landeira, D., Sauer, S., Poot, R., Dvorkina, M., Mazzarella, L., Jørgensen, H.F., Pereira, C.F., Leleu, M., Piccolo, F.M., Spivakov, M., et al. (2010). Jarid2 is a PRC2 component in embryonic stem cells required for multi-lineage differentiation and recruitment of PRC1 and RNA Polymerase II to developmental regulators. Nat. Cell Biol. *12*, 618–624.

Li, X., Isono, K.-I., Yamada, D., Endo, T.A., Endoh, M., Shinga, J., Mizutani-Koseki, Y., Otte, A.P., Casanova, M., Kitamura, H., et al. (2011). Mammalian polycomb-like Pcl2/Mtf2 is a novel regulatory component of PRC2 that can differentially modulate polycomb activity both at the Hox gene cluster and at Cdkn2a genes. Mol. Cell. Biol. *31*, 351–364.

van Mierlo, G., Wester, R.A., and Marks, H. (2019). A Mass Spectrometry Survey of Chromatin-Associated Proteins in Pluripotency and Early Lineage Commitment. Proteomics *19*, e1900047.

Motenko, H., Neuhauser, S.B., O'Keefe, M., and Richardson, J.E. (2015). MouseMine: a new data warehouse for MGI. Mamm. Genome *26*, 325–330.

Perino, M., van Mierlo, G., Karemaker, I.D., van Genesen, S., Vermeulen, M., Marks, H., van Heeringen, S.J., and Veenstra, G.J.C. (2018). MTF2 recruits Polycomb Repressive Complex 2 by helical-shape-selective DNA binding. Nat. Genet. *50*, 1002–1010.

Ramírez, F., Ryan, D.P., Grüning, B., Bhardwaj, V., Kilpert, F., Richter, A.S., Heyne, S., Dündar, F., and Manke, T. (2016). deepTools2: a next generation web server for deep-sequencing data analysis. Nucleic Acids Res. *44*, W160–W165.

Schoeftner, S., Sengupta, A.K., Kubicek, S., Mechtler, K., Spahn, L., Koseki, H., Jenuwein, T., and Wutz, A. (2006). Recruitment of PRC1 function at the initiation of X inactivation independent of PRC2 and silencing. EMBO J. *25*, 3110–3122.

Zhang, Y., Liu, T., Meyer, C.A., Eeckhoute, J., Johnson, D.S., Bernstein, B.E., Nussbaum, C., Myers, R.M., Brown, M., Li, W., et al. (2008). Model-based Analysis of ChIP-Seq (MACS). Genome Biol. *9*, R137.

Zhou, T., Yang, L., Lu, Y., Dror, I., Dantas Machado, A.C., Ghane, T., Di Felice, R., and Rohs, R. (2013). DNAshape: a method for the high-throughput prediction of DNA structural features on a genomic scale. Nucleic Acids Res. *41*, W56–W62.
